# Improving the understanding of how patients with non-dystrophic myotonia are selected for myotonia treatment with mexiletine (NaMuscla): outcomes of treatment impact using a European Delphi panel

**DOI:** 10.1186/s12883-021-02491-3

**Published:** 2021-12-01

**Authors:** Ann-Marie Chapman, Marieke Schurer, Laure Weijers, Amer Omar, Hiba Lee, Alla Zozulya Weidenfeller, Crispin Ellis, Shaneil Sonecha, Christiane Schneider-Gold

**Affiliations:** 1 BresMed Health Solutions Ltd, Sheffield, UK; 2 BresMed Netherlands BV, Utrecht, The Netherlands; 3 Lupin Atlantis Holdings SA, Zug, Switzerland; 4 AGH Partners Ltd, London, UK; 5 Lupin Healthcare Ltd, Slough, UK; 6 St. Josef-Hospital, Bochum, Germany

**Keywords:** Non-dystrophic myotonia, Delphi panel, Mexiletine (NaMuscla), Quality of life, Healthcare resource utilisation, INQoL, EQ-5D

## Abstract

**Background:**

Non-dystrophic myotonias (NDMs) comprise muscle chloride and sodium channelopathies due to genetic defects of the CLCN1- and SCN4A-channels. No licensed antimyotonic treatment has been available until approval of mexiletine (NaMuscla®) for adult patients by the EMA in December 2018. This Delphi panel aimed to understand how outcomes of the pivotal phase III Mexiletine study (MYOMEX) translate to real world practice and investigate health resource use, quality of life and the natural history of NDM to support economic modelling and facilitate patient access.

**Methods:**

Nine clinical experts in treating NDM took part in a two-round Delphi panel. Their knowledge of NDM and previous use of mexiletine as an off-label treatment prior to NaMuscla’s approval ensured they could provide both qualitative context and quantitative estimates to support economic modelling comparing mexiletine (NaMuscla) to best supportive care. Consensus in four key areas was sought: healthcare resource utilization (HRU), treatment with mexiletine (NaMuscla), patient quality of life (QoL), and the natural history of disease. Concept questions were also asked, considering perceptions on the feasibility of mapping the validated Individualized Neuromuscular Quality of Life (INQoL) instrument to the generic EQ-5D™, and the potential impact on caregiver QoL.

**Results:**

Consensus was achieved for key questions including the average long-term dosage of mexiletine (NaMuscla) in practice, the criteria for eligibility of myotonia treatment, the clinical importance of QoL outcomes in MYOMEX, the higher proportion of patients with increased QoL, and the reduction in the need for mental health resources for patients receiving mexiletine (NaMuscla). While consensus was not achieved for other questions, the results demonstrated that most experts felt mexiletine (NaMuscla) reduced the need for HRU and was expected to improve QoL. The QoL mapping exercise suggested that it is feasible to map domains of INQoL to EQ-5D. Points of interest for future research were identified, including that mexiletine (NaMuscla) may slow the annual decrease in QoL of patients over their lifetime, and a significant negative impact on QoL for some caregivers.

**Conclusions:**

This project successfully provided data from an informed group of clinical experts, complementing the currently available clinical trial data for mexiletine (NaMuscla) to support patient access decisions.

**Supplementary Information:**

The online version contains supplementary material available at 10.1186/s12883-021-02491-3.

## Background

Non-dystrophic myotonias (NDMs) are a heterogeneous group of genetic diseases caused by mutations in the genes coding for skeletal muscle chloride (*CLCN1*) and sodium ion channels (*SCN4A*) [1–3]. NDM is a very rare disorder; while robust evidence is lacking and the epidemiology varies per country, the prevalence of NDM is estimated to be 1:100,000 people in the European Union (EU) taking all subtypes together [4, 5].

Symptoms of NDM typically present in childhood, often in the first decade of life [2]. The major clinical manifestation is muscle stiffness or locking, caused by myotonia, but depending on the subtypes of NDM, patients may also experience muscle weakness, pain and fatigue [2, 3]. Muscle weakness can vary in severity. Depending on the type of mutation/channelopathy, it can be transient or episodic in nature. In paramyotonia (weakness triggered by cold and physical effort) and in autosomal recessive chloride channel myotonia (weakness occurring and resolving during continuous repetitive movements), muscle weakness typically lasts seconds to minutes [2]. In contrast, in hyperkalaemic period paralysis, weakness occurs in episodes lasting hours to days, with some patients experiencing marked tetraparesis [2, 6]. Patients with NDM may also feel functionally weak due to their myotonia and the exertion needed to overcome myotonic stiffness [7]. In some severe cases, impaired mobility, risk of falling and affected speech have been reported [8]. While NDM does not lead to mortality, it is a chronic debilitating condition associated with life-long symptoms and a negative impact on physical functioning [4, 9, 10]. Several studies have reported that myotonia (muscle stiffness) drastically reduces the patient’s ability to perform daily activities [8, 10] and that symptomatic patients suffer from a diminished quality of life (QoL) [9–11]. While much is still unknown about the progression of the disease, Trip et al. [12] found in a cross-sectional study that the majority of patients with NDM will experience an increase of symptoms over their lifetime.

Historically, medications of various pharmacological classes have been administered (off-label) in patients with symptomatic myotonia [13, 14]. Mexiletine (NaMuscla®), a sodium channel antagonist, is the first and only medicinal product currently approved in the EU for the symptomatic treatment of myotonia in adult patients with NDM [15]. The Efficacy and Safety of Mexiletine (NaMuscla) in Nondystrophic Myotonias (MYOMEX) trial showed that treatment with mexiletine (NaMuscla) resulted in a significant reduction in median self-reported stiffness visual analogue scale (VAS) score, as well as an improvement in QoL as measured using the Individualized Neuromuscular Quality of Life Questionnaire (INQoL) [16, 17].

Despite the variability in pathophysiology of chloride and sodium channelopathies, mexiletine exhibits a similar mode of action and efficacy in these various subtypes. For chloride channelopathies, mexiletine has been shown to be an effective and safe anti-myotonic treatment after investigation in at least 120 patients, over a treatment duration of at least 8 years [16, 18–25]. Similarly, mexiletine has been shown to be effective and safe in reducing myotonia in patients with sodium channelopathies, based on investigation in at least 63 patients including patients with sodium channel mutations [16, 21], patients with paramyotonia congenita [26–28], myotonia fluctuans [23, 29] and hyperPP [30]. In addition, current NDM treatment guidelines recommend the use of mexiletine in both types of channelopathies [2, 31].

Although the evidence for mexiletine (NaMuscla) supported regulatory approval, evidence gaps exist that could impede discussions with payers around national-level patient access. This includes discussions on how to generalize the clinical trial outcomes to national-level clinical practice, how to quantify the impact of NDM on QoL using payer-preferred QoL instruments to allow QoL incorporation into economic modelling, and how mexiletine (NaMuscla) compares with best supportive care (BSC) in terms of healthcare resource utilization (HRU).

With rising health expenditure due to aging populations and the increasing availability of new, expensive technologies, healthcare decision-makers are faced with the challenging task of allocating scarce resources to maximise health benefits for the population as a whole. Many European countries require some form of health technology assessment (HTA), including an economic evaluation, to inform decision makers about the costs and clinical benefits of an intervention and allow them to make informed reimbursement decisions. This is typically evidenced by comparing the cost per quality-adjusted life year (QALY) of the intervention with that of the current standard of care [32]. The QALY is a measure that incorporates length of life along with health-related QoL using utility values; a cardinal value that reflects an individual’s preference for different health states ranging from zero (reflecting states of health equivalent to death) to one (reflecting perfect health) [33]. While many QoL instruments exist, HTA decision-makers generally prefer the use of the EQ-5D – a generic QoL instrument that allows comparison of QoL across diseases. If EQ-5D data is not available, it is largely recommended to map outcomes of conditionspecific instruments to the EQ-5D, if possible [34].

To be able to meet HTA decision-makers’ needs and develop an economic model for mexiletine (NaMuscla) in NDM, sufficient information is needed to inform the model. This includes data on the costs associated with the resource use of patients with NDM, as well as the clinical benefits and QoL outcomes of patients with NDM who are treated with mexiletine (NaMuscla) versus those who only receive BSC. A substantial limitation in the current evidence base for mexiletine (NaMuscla) is the lack of published literature describing the healthcare resources needed by patients with NDM and how often these resources are used, data that an economic model requires to project costs. In addition, the MYOMEX trial measured QoL using INQoL. Even though a condition-specific QoL instrument such as INQoL is more likely to capture the full impact of a condition on QoL than a generic measure [11, 35], the INQoL does not have a utility-based scoring algorithm and therefore cannot directly be used to measure QALYs. Moreover, there is currently no established algorithm to map the INQoL to the EQ-5D, which poses challenges in the use of QoL data from the trial in economic evaluations.

Given ongoing discussions with patient access decision makers across Europe, there is an immediate need for additional information to complement the available evidence from the clinical trials. Therefore, this study’s objective was to use a Delphi panel to:

- Investigate the healthcare resource utilization of adult patients with NDM who are being treated with mexiletine (NaMuscla) compared with patients who receive BSC, from the perspective of the UK National Health Service (NHS) and Personal Social Services
- Explore the concept of mapping domains of the INQoL to domains of the EQ-5D to independently verify a conceptual matching exercise completed in a previous study conducted by Lloyd et al. [36] to develop appropriate utility measures
- Better understand how NDM may progress over time in adult patients, in terms of patients’ QoL
- Explore the potential impact of NDM on caregivers’ QoL

## Methods

### Study design

An online two-round Delphi panel study was conducted between 18 March and 27 July 2020 (note: the study duration was prolonged due to the exceptional circumstances of the COVID-19 pandemic) using SurveyMonkey®. The Delphi method was considered well suited to the aims of this project, as it is a widely used and accepted method for achieving convergence of opinion concerning real-world knowledge solicited from experts, and can support decision making by collecting data in a relatively timely manner. One of the key strengths of the Delphi method includes quasi-anonymity (i.e. the panellists may know the identities of the other panellists, but the responses are not assigned to individual panellists), which avoids dominance of one opinion; and iteration, which allows individuals to change their opinions based on the information and explanations provided by other panellists in previous rounds [37].

### Development of study materials

Study materials, including a project plan, pre-read materials for the experts and the first round Delphi questionnaire were developed by two BresMed researchers (MS and LW). The project plan outlined the research questions, study methods, a recruitment plan, and an analysis plan. Questionnaire themes were based on a previously conducted literature review (data on file), which identified data gaps on the HRU of patients with NDM in the UK and the natural history of NDM. In addition, questions were added to obtain information to support important economic model assumptions and discussions with decision makers, such as validation of QoL results of clinical trials, exploration of the ability to map the INQoL to the EQ-5D, potential impact on caregiver QoL, and understanding what aspects of NDM impact patient QoL most.

All study materials were reviewed by the project leads from BresMed (AMC) and the study sponsor (AO, AZW, CE, HL and SC), and an independent medical expert in the field of myotonic disorders, Professor Schneider-Gold (CSG). Two BresMed researchers who were not involved in this study piloted the questionnaire to test its functionality and clarity. Finally, the questionnaire was piloted and reviewed by CSG, to ensure appropriateness of the content and minimize the risk of bias. The final Round 1 (RD1) questionnaire consisted of six main sections and a mix of 30 open-ended and closed questions (Additional file 1). The questionnaire was designed so it could be completed in 1 h. The second questionnaire followed a similar structure as the first questionnaire; analysed results of RD1 were presented back to the panellists, and follow-up questions were included to move towards a consensus (Additional file 2).

### Expert selection

Selection of appropriate subjects is the most important step in the Delphi process, as this will have a large influence on the quality of the results generated [37]. Therefore, purposive sampling was carried out to invite experts who were considered most suitable to address the research problem [38].

Due to the rarity of the disease, a target maximum of 10 experts were sought. As it was a priority to obtain UK data on HRU for the economic model, most experts were from the UK: a total of six UK medical experts were identified and invited to participate in this study. In addition, one expert each from Denmark, Germany and France were invited, to understand the generalizability of the results across European countries. The study sponsor was responsible for contracting experts, based on pre-specified selection criteria (Additional file 3) to reduce bias and ensure an appropriate level of experience in NDM, experience with mexiletine (NaMuscla), and overall representativeness of the panel.

### Data analysis

Open-ended questions were analysed thematically. Closed questions were analysed using measures of central tendency such as means, median and mode, and levels of dispersion (ranges) when appropriate. The threshold for consensus was pre-specified as 70% agreement among the panellists. Data were processed as ‘intention-to-participate’, such that the consensus threshold was based on the initial number of panellists who agreed to participate in that respective Delphi round, regardless of the number lost to follow-up. An exception to this were the questions relating to HRU. Because HRU is country specific, responses to the economic section collated answers that were UK specific and the intent-to-participate population was specific to the UK.

All questions were mandatory except for open-ended questions that aimed to collect additional data. However, panellists were given the option to note if they were unsure about the answer or if the question was irrelevant to their role. The data was analysed by MS and LW and reviewed by AMC. Any possible errors or mistakes were followed up by MS and LW with the experts individually, to improve accuracy and completeness of the responses. The overall results were presented to the study sponsor, but the sponsor was not involved in the analysis and did not have access to the individual-level data.

The final study report was shared with all panellists for any final remarks; however, no additional comments were received.

### Ethics

This research was conducted in accordance with applicable national standards and regulations. This study was not conducted on humans, nor did it involve elicitation of patients, analysis of direct patient data, or research on human tissue/DNA. In addition, because all participants in this Delphi panel are adult health care professionals who were contracted by the study sponsor, no ethics approval was required according to the UK Health Research Authority (HRA) guideline [39]. The experts’ contracts outlined the aims of the study, the methods, and information about how their data would be used.

Informed consent was obtained from all study participants to i) participate in this study and ii) use their anonymised data in publications. In addition, informed consent was obtained from panellists from Denmark, France and Germany to use their non-anonymised responses on HRU. All panellists received financial compensation for their time from the study sponsor.

## Results

Nine experts were identified, contracted and invited to participate in this study, as detailed in Table 1 (UK: 6, FR: 1, DE: 1, DK: 1); eight completed RD1 (response rate 89%). All calculations of RD1 were based on an intention-to-participate population of nine, except for the HRU section, which was analysed per country. Similarly, one further expert from the UK dropped out in RD2, therefore only seven out of eight experts provided responses (response rate 87.5%). All calculations for RD2 were therefore based on an intention-to-participate population of eight, apart from the HRU section. The experts participating in RD1 had on average over 17 years’ experience in treating patients with NDM. All experts are considered key opinion leaders in the field of NDM, through contributing to the scientific community (e.g. in the form of publications, speaking at relevant conferences, and/or participation in neuromuscular research societies), their experience in managing NDM patients, and their clinical involvement (e.g. leading clinical trials, other clinical research and/or as member of neuromuscular expert commissions).

**Table 1:** Characteristics of the responding panellists

Country	Role	Years’ experience managing patients with NDM	Number of patients with NDM registered in centre/hospital	Current NDM patients in expert care^**a**^	Experience with mexiletine (NaMuscla)
France	Neurologist	> 15 years	> 50	11–20	Yes
UK	Neurologist^b^	> 10 years	5–10	< 5	Yes
UK	Clinical geneticist	≥ 20 years	5–10	< 5	Yes
Denmark	Neurologist	≥ 20 years	> 50	21–50	Yes
UK	Neurologist	> 10 years	5–10	5–10	Yes
UK	Specialist nurse	1–5 years	11–20	11–20	Yes
UK	Neurologist	≥ 20 years	11–20	5–10	Yes
Germany	Neurologist	≥ 20 years	> 50	21–50	Yes

Only participants responding to at least Round 1 are included in this table

Key: *NDM* Non-dystrophic myotonia

^a^Please note that NDM is a rare disease, therefore the number of patients under the panel’s care at any one time is expected to be relatively low. Moreover, the number of patients currently under the care of our expert panel is a snapshot indicator of the experts’ experience with treating NDM. All experts have treated substantially more patients with NDM over the duration of their career

^b^This expert dropped out after completing Round 1

### Healthcare resource utilization

The first round of the Delphi panel aimed to obtain mean values of pre-specified categories of resource use, identify important additional missing resources, and understand potential differences between patients who receive BSC and those receiving mexiletine (NaMuscla). BSC was defined as any supportive care that symptomatic adult patients with NDM may receive that does not involve symptom-modifying pharmacological treatment. This includes, for example, the use of supportive medication (e.g. pain killers), mobility aids, physiotherapy or speech therapy. Tables 2 and 3 give an overview of the estimated proportion of UK patients with NDM requiring each resource and the frequency of this resource use, respectively.

**Table 2:** Proportion of patients with NDM requiring resources in the UK

Health resource^a^	Round 1 (***n*** = 5, 1 drop-out)				Round 2 (***n*** = 4, 1 drop-out)
	BSC – Mean % of patients requiring resource (range)	Mexiletine – Mean % of patients requiring resource (range)	Difference (%)	BSC:mexiletine ratio^**b**^	% Agreement that HRU is on average lower for patients treated with mexiletine among panel (% agreement among responders)
Physiotherapy	39.0 (15–60)	23.0 (5–60)	−16.0	1.7	60% (75%)
Occupational therapist	15.0 (5–25)	6.0 (0–15)	−9.0	2.5	60% (75%)
Speech therapy	5.0 (0–15)	1.0 (0–5)	−4.0	5.0	40% (50%)
**Day case attendances**	**60.0 (0–100)**	**60.0 (0–100)**	**0.0**	**0**	**NA**
**Wheelchair**	**4.2 (0–20)**	**4.2 (0–20)**	**0.0**	**0**	**NA**
Walking stick	7.2 (0–30)	2.2 (0–10)	−5.0	3.3	60% (75%)
Walking frame	0.4 (0–2)	0.0 (0–0)	−0.4	0	60% (75%)
Hospital admission for fracture	3.0 (0–10)	1.0 (0–5)	−2.0	3.0	60% (75%)
Overall BSC:mexiletine (NaMuscla) ratio				1.9	60% (75%)
**Mental health support**	**38 (20–60)**	**14 (5–20)**	**−24**	**2.7**	**83% (100%)**

Key: *BSC* Best supportive care (defined as any supportive care that symptomatic adult patients with NDM may receive that does not involve symptom-modifying pharmacological treatment; includes, for example, the use of supportive medication [e.g. pain killers], mobility aids, physiotherapy or speech therapy), *HRU* Healthcare resource utilization, *NDM* Non-dystrophic myotonia

^a^The rows in bold indicate resources for which there was consensus

^b^The ratio was calculated by dividing the mean proportion of patients receiving BSC that require a resource by the mean proportion of patients treated with mexiletine (NaMuscla) that require the resource

**Table 3:** Frequency of resource use per patient with NDM per year in the UK^a^

Health resource	Round 1 (***n*** = 5, 1 drop-out)				Round 2 (***n*** = 4, 1 drop-out)
	BSC – Mean number of annual visits (range)	Mexiletine – Mean number of annual visits (range)	Difference (%)	BSC:mexiletine ratio^**b**^	% Agreement that HRU is on average lower for patients treated with mexiletine among panel (% agreement among responders)
Physiotherapy	5.0 (2–9)	4.0 (0–9)	−1.0	1.3	60% (75%)
Occupational therapist	2.2 (1–5)	1.2 (0–4)	−1.0	1.8	60% (75%)
Speech therapy	1.2 (0–5)	0.4 (0–2)	−0.8	3.0	40% (50%)
Day case attendances	1.0 (0–2)	0.8 (0–2)	−0.2	1.3	40% (50%)
Overall BSC:mexiletine (NaMuscla) ratio				1.8	60% (75%)

Key: *BSC* Best supportive care (defined as any supportive care that symptomatic adult patients with NDM may receive that does not involve symptom-modifying pharmacological treatment; includes, for example, the use of supportive medication [e.g. pain killers], mobility aids, physiotherapy or speech therapy), *HRU* Healthcare resource utilization, *NDM* Non-dystrophic myotonia

^a^Please note that mobility aids are not included in this table as it was assumed that patients requiring this resource would only need one walking frame, wheelchair and/or walking stick per year

^b^The ratio was calculated by dividing the mean number of annual visits of patients receiving BSC that require a resource by the mean number of annual visits of patients treated with mexiletine (NaMuscla) that require the resource

As can be seen, the estimations varied considerably. However, there was consensus among the UK experts (83%; 5/6) that there is no difference between patients receiving BSC and mexiletine (NaMuscla) in the proportion of patients requiring day case attendances or a wheelchair. Results of the other resources suggested that on average, the proportion of patients making use of any resource is lower for patients treated with mexiletine (NaMuscla) compared with patients receiving BSC, with the proportion of patients on BSC that require any resource being 1.9 times higher than the proportion of patients who receive mexiletine (NaMuscla) (Table 2). On the question about whether any resources were missing from the list of pre-specified resources, three experts noted that patients may require help from an ‘NDM family care advisor’ (*n* = 1), ‘social worker’ (*n* = 1), or a ‘muscular dystrophy advocacy officer’ (*n* = 1).

In RD2, the panel was asked to confirm the hypothesis that the proportion of patients requiring each individual category of HRU would on average be lower for patients treated with mexiletine (NaMuscla). While no consensus was achieved, the majority of UK experts (60%; 3/5) agreed with this statement for all resources except speech therapy (40%; 2/5). Similarly, 60% (3/5) agreed that a resource multiplier of 1.9 for those receiving BSC is a reasonable reflection of the difference in the proportion of patients requiring HRU in the UK.

When looking at the number of healthcare visits, similar outcomes were found. The results suggested that, on average, the frequency of healthcare visits of patients who make use of a particular resource is lower when patients receive mexiletine (NaMuscla) compared with patients receiving BSC. Looking at the overall difference, the number of visits for patients requiring any resource is 1.8 times higher when receiving BSC compared with those treated with mexiletine (NaMuscla). In RD2, 60% (3/5) of the UK experts agreed with the statement that the number of healthcare visits related to physiotherapy and occupational therapy would be lower for patients receiving mexiletine (NaMuscla), whereas only 40% (2/5) felt this was the case for day case attendances and visits related to speech therapy. The majority of UK experts (60%; 3/5) agreed in RD2 that the number of visits for patients with NDM is on average 1.8 times higher for patients who receive BSC compared with those treated with mexiletine (NaMuscla).

With respect to mental health support in general, consensus among UK experts was achieved that the proportion of patients requiring this resource is lower for patients who are treated with mexiletine (NaMuscla) compared with those receiving BSC (83%; 5/6) in RD1. This difference averaged out as 2.7 times more mental health support being needed for patients receiving BSC. When asked about the type of mental health resources patients with NDM may use, the panel identified a range of services, which can be summarised as care from a: psychiatrist, neuropsychologist, general practitioner, psychologist and patient support organisation.

In RD2 the results were presented back to the panellists, with the majority of UK experts (60%; 3/5) agreeing in this round that the need for mental health support is on average 2.7 times higher for patients receiving BSC than for those receiving mexiletine (NaMuscla). In addition, the experts provided estimates on the proportion of patients with NDM that require each identified mental health resource and the frequency of visits (Tables 4 and 5). Interestingly, the estimates were expected to be nearly the same for patients receiving BSC and those receiving treatment with mexiletine (NaMuscla), except for care provided by the general practitioner, which is expected to be lower for those receiving treatment. One expert pointed out that the *need* for mental health support might not correspond with the actual HRU:

*“Please note mental health is vastly under-resourced in my region. My answers do not reflect a need which is far far higher.”* (C3)

**Table 4:** Proportion of patients with NDM requiring mental health support in the UK

Mental health resource	BSC – Mean % of patients requiring resource (range)	Mexiletine – Mean % of patients requiring resource (range)	Difference (%)	BSC:mexiletine ratio^**a**^
Neuropsychologist	0 (0)	0 (0)	0	1
General practitioner (mental health-related visits)	42.5 (0-100)	37.5 (0-100)	-5	1.1
Psychiatrist	2.5 (0-10)	2.5 (0-10)	0	1
Psychologist	3.75 (0-10)	3.75 (0-10)	0	1

Four experts completed this question in RD2, one expert dropped-out

Key: *BSC* Best supportive care (defined as any supportive care that symptomatic adult patients with NDM may receive that does not involve symptom-modifying pharmacological treatment; includes, for example, the use of supportive medication [e.g. pain killers], mobility aids, physiotherapy or speech therapy), *HRU* Healthcare resource utilization

^a^The ratio was calculated by dividing the mean proportion of patients receiving BSC that require a resource by the mean proportion of patients treated with mexiletine (NaMuscla) that require the resource

**Table 5:** Frequency of mental health resource use per patient with NDM per year in the UK

Mental health resource	BSC – Mean number of annual visits (range)	Mexiletine – Mean number of annual visits (range)	Difference (%)	BSC:mexiletine ratio^**a**^
Neuropsychologist	0 (0-0)	0 (0-0)	0	1
General practitioner (mental health-related visits)	7.25 (0-20)	5.25 (0-12)	-2	1.4
Psychiatrist	0.5 (0-2)	0.5 (0-2)	0	1
Psychologist	4 (0-10)	3 (0-6)	-1	1.3

Four experts completed this question in RD2, one expert dropped-out

Key: *BSC* Best supportive care (defined as any supportive care that symptomatic adult patients with NDM may receive that does not involve symptom-modifying pharmacological treatment; includes, for example, the use of supportive medication [e.g. pain killers], mobility aids, physiotherapy or speech therapy), *HRU* Healthcare resource utilization

^a^The ratio was calculated by dividing the mean proportion number of annual visits of patients receiving BSC that require a resource by the mean proportion number of annual visits of patients treated with mexiletine (NaMuscla) that require the resource

Tables 6 and 7 provide an overview of the results regarding HRU and the frequency of HRU in France, Germany and Denmark. While the results are based on only one expert per country, they seem to underline that the categories of HRU are similar across the countries. However, the use of these resources is country-specific. The impact of mexiletine (NaMuscla) use on the proportion of patients using resources appears to vary across the countries – for every resource a reduction in the proportion of patients requiring that resource was suggested in France; for Germany, the results suggested no change, while in Denmark the impact ranged from a reduction to an increase in resource use. This demonstrates the need to collect HRU data on a country level.

**Table 6:** Proportion of patients with NDM requiring resources in France, Germany and Denmark

Health resource	France (%)			Germany (%)			Denmark (%)		
	BSC	Mexiletine	Difference	BSC	Mexiletine	Difference	BSC	Mexiletine	Difference
Physiotherapy	40	10	−30	100	100	0	25	25	0
Occupational therapist	20	10	−10	20	20	0	15	15	0
Speech therapy	30	5	−25	20	20	0	5	0	−5
Day case attendances	100	50	−50	100	100	0	5	0	−5
Use of wheelchair	10	0	−10	1	1	0	0	5	+ 5
Use of walking stick	20	10	−10	10	10	0	10	0	−10
Use of walking frame	10	5	−5	1	1	0	0	0	0
Hospital admission for fracture	5	2	−3	1	1	0	0	0	0

Estimates are based on one expert per country

Key: *BSC* Best supportive care

**Table 7:** Frequency of resource use per patient with NDM per year in France, Germany and Denmark

Health resource	France			Germany			Denmark		
	BSC	Mexiletine	Difference	BSC	Mexiletine	Difference	BSC	Mexiletine	Difference
Physiotherapy	30	5	−25	24	24	0	40	40	0
Occupational therapist	10	2	−8	12	12	0	6	6	0
Speech therapy	15	2	−13	12	12	0	6	0	−6
Day case attendances	5	1	−4	4	4	0	6	0	−6

Whenever a 0 is presented, this reflects that the expert reported that they expect 0 healthcare professional visits for that resource

Key: *BSC* Best supportive care

### Treatment in clinical practice

In the MYOMEX trial, patients received a starting dose of 200 mg (i.e. one capsule) mexiletine hydrochloride (HCl) per day (i.e. one capsule equivalent to 167 mg mexiletine [NaMuscla]), which was gradually increased to a maximum of 600 mg daily (i.e. three capsules equivalent to 500 mg mexiletine [NaMuscla]) [40]. However, a retrospective review from Suetterlin et al. [21] showed that the mean effective daily dose in their clinical centre varied between 333 mg and 550 mg mexiletine HCl, equating to 400 mg (i.e. two capsules equivalent to 333 mg mexiletine [NaMuscla]) to 600 mg (i.e. three capsules equivalent to 500 mg mexiletine) per day. To gain more insight into the dosage and prescription criteria in real life, the panel was asked about the average long-term daily dosage and the criteria used to select patients for treatment with mexiletine (NaMuscla).

Consensus was reached in RD1 that, on average, two capsules of mexiletine (NaMuscla) would be taken daily by the general adult NDM patient population (78%; 7/9) in the long term. In RD2, it was explored whether the dosage would be the same, lower, or higher for patients aged > 65 years old. While no consensus was achieved on a single answer option, all responding experts (88%, 7/8, one drop-out) believed the average daily dose to be either two capsules or less for this older patient population. Two experts pointed out that tolerance and co-morbidities might play a bigger role in considering treatment with mexiletine (NaMuscla):

*‘[…] in reality [I] would expect very few patients to request treatment beyond 65. I would be more concerned about a higher chance of having contraindications’ (C3)*

*‘Tolerance expected to be lower than <65y adults due to age, co-morbidities and other drugs’ (C1)*

**Fig. 1:**
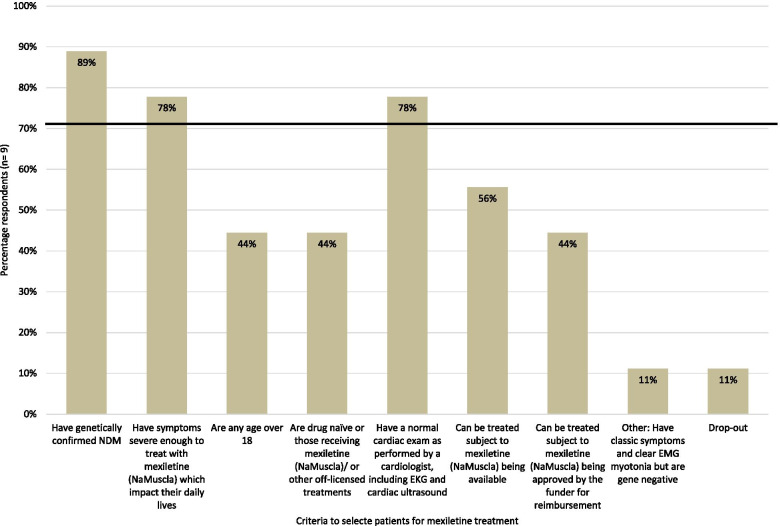
Criteria to select patients with NDM for mexiletine (NaMuscla) treatment. Key: EKG, electrocardiogram; EMG, electromyographic; NDM, non-dystrophic myotonia. Note: The horizontal black line indicates the 70% consensus threshold

Seven prescription criteria were presented to the panel, based on inclusion criteria used in the MYOMEX trial (i.e. ‘genetically confirmed NDM’, ‘symptoms severe enough to treat, which impact their daily live’, ‘any age over 18’, ‘drug naïve, or receive mexiletine (NaMuscla), or other offlicence treatments’, ‘normal cardiac exam as performed by a cardiologist, including EKG [electrocardiogram] and cardiac ultrasound’) and criteria related to the accessibility of treatment (i.e. ‘subject to NaMuscla [mexiletine (NaMuscla)] being available’, ‘subject to NaMuscla [mexiletine (NaMuscla)] being approved by the funder for reimbursement’). Of these, consensus was reached on three criteria (Fig. 1). Experts agreed they would consider treatment with mexiletine (NaMuscla) if patients have genetically confirmed NDM (89%; 8/9), symptoms severe enough to treat with mexiletine (NaMuscla) that impact their daily life (78%; 7/9) and/or have a normal cardiac exam as performed by a cardiologist, including EKG and cardiac ultrasound (78%; 7/9). No consensus was reached on the remaining four selection criteria. One expert noted they would also consider someone for treatment with mexiletine (NaMuscla) if they ‘have classic symptoms and clear EMG [electromyographic] myotonia but are gene negative’.

### Quality of life

In the MYOMEX trial, a statistically significant improvement was found in the absolute change in median stiffness VAS score and absolute mean change in INQoL from baseline. To validate these findings from a clinical perspective, experts were presented with the stiffness VAS and overall INQoL scores of the trial and asked if they believed the scores to be clinically meaningful. Consensus was achieved in RD1 on both aspects: 78% (7/9) and 89% (8/9) of the experts noted that the absolute change in median stiffness VAS score and the absolute mean change in overall QoL of the INQoL in the mexiletine (NaMuscla) arm from baseline are clinically meaningful changes, respectively.

Health technology agencies who consider cost effectiveness may look to incorporate QoL as measured by the generic EQ-5D, which provides a single utility measure rather than information over a range of QoL domains. Because the MYOMEX trial captured QoL using the INQoL and there is no validated algorithm to map INQoL to EQ-5D, part of this study aimed to explore how the experts felt the domains and items of INQoL might map to EQ-5D.

In RD1, consensus was achieved on three of the five domains of EQ-5D (Table 8). All responding experts mapped ‘usual activities’ to ‘the things you do – leisure and work activities’ from INQoL (89%, 8/9, one drop-out), ‘pain/discomfort’ to ‘your pain’ (89%, 8/9, one drop-out), and most experts mapped ‘anxiety/depression’ to ‘how you feel/emotions’ (78%, 7/9). No consensus was reached for the ‘mobility’ and ‘self-care’ domains of the EQ-5D. When following up on those two domains in RD2, there was consensus among the panel (75%, 6/8) that ‘self-care’ has most overlap with the question ‘At the moment, does your muscle condition affect your ability to do the following activities: daily activities (for example, washing, dressing and housework)’ of the ‘things you do’ domain of INQoL. While no consensus was achieved on the ‘mobility’ domain in RD2, the expert responses clearly demonstrated that mobility for NDM is a mix of muscle weaknesses (50%, 4/8) and muscle locking (37.5%, 3/8), with no one aspect dominating the other.

**Table 8:** Matching INQoL Domains to EQ-5D

ED-5Q domain	INQoL domain^**a**^	CR1	% agreement among panel (% of responders)^**b**^	CR2	% agreement among panel (% of responders)^**c**^
**Usual activities**	**The things you do – leisure and work activities**	**Yes**	**89% (100%)**	**NA**	**NA**
**Pain/discomfort**	**Your pain**	**Yes**	**89% (100%)**	**NA**	**NA**
**Anxiety/depression**	**How you feel/emotions**	**Yes**	**78% (87.5%)**	**NA**	**NA**
Mobility	The locking of your muscles	No	44% (50%)	No	37.5% (43%)
	Your muscle weakness	No	55% (62.5%)	No	50% (71%)
	How tired you feel/fatigue	No	22% (25%)	NA	NA
	Your independence	No	11% (12.5%)	NA	NA
	The way you look/body image	No	11% (12.5%)	NA	NA
**Self-care (washing and dressing)**	The things you do – daily activities	No	44% (50%)	NA	NA
	**At the moment, does your muscle condition affect your ability to do the following activities: daily activities (for example, washing, dressing and housework)**	**NA**	**NA**	**Yes**	**75% (86%)**
	Your independence	No	44% (50%)	NA	NA
	How important to you is the effect of your muscle condition on your level of independence	NA	NA	No	12.5% (14%)
	The way you look/body image	No	11% (12.5%)	NA	NA

The rows highlighted in bold indicate domains for which consensus was reached

Key: *CR1* Consensus Round 1, *CR2* Consensus Round 2, *INQoL* Individualized Neuromuscular Quality of Life Questionnaire, *NA* Not applicable

^a^Experts could map multiple domains of the INQoL to one domain of the EQ-5D, however, they could map each unique domain of the INQoL only once

^b^Eight of the nine invited experts completed round one of the Delphi; one expert dropped out

^c^Seven of the eight invited experts completed round two of the Delphi; one expert dropped out

**Fig. 2:**
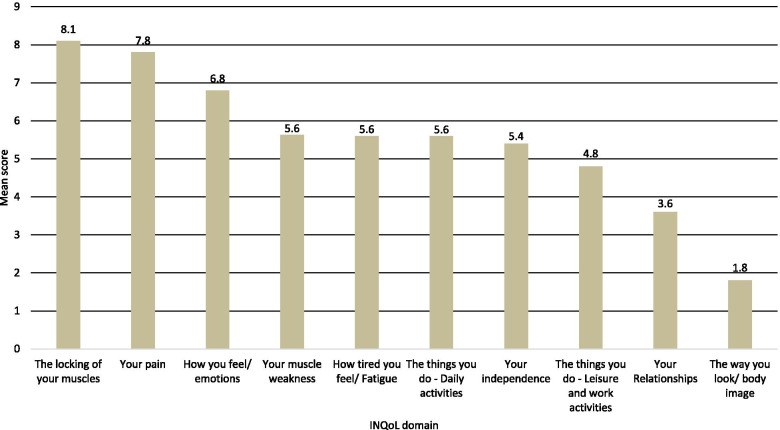
Perceived impact of INQoL domains on patient QoL. Key: INQoL, Individualized Neuromuscular Quality of Life; QoL, quality of life. Note: Higher scores indicate higher perceived impact on patient QoL. Experts were asked to rank domains of INQoL based on their perceived impact on QoL (from 1 – highest impact, to 10 – lowest impact). The overall score for each domain was derived by calculating the mean of all expert responses (assigning the most impactful domain a 10 and the least impactful domain a 1)

Finally, this section of the Delphi study aimed to understand which domains of the INQoL are perceived to impact patients’ QoL most as this can provide valuable insights into treatment objectives, without the desire to achieve a consensus on this question. Figure 2 presents the results. Overall, ‘the locking of your muscles’ was ranked across the panel as the most impactful aspect (mean score 8.1), followed by ‘your pain’ (7.8) and ‘how you feel/emotions’ (6.8).

### Natural history of the disease

While much is still unknown about the natural history of NDM, the study from Trip et al. [9] suggested that NDM substantially affects the patients’ physical health status, with painful myotonia and fatigue being the strongest predictors of the deficits over time. We explored the hypothesis by investigating the proportion of patients who would experience an increase, decrease or no change in their QoL over their lifetime (RD1) and the rate at which this might differ, both qualitatively (RD1) as well as quantitatively (RD2).

**Fig. 3:**
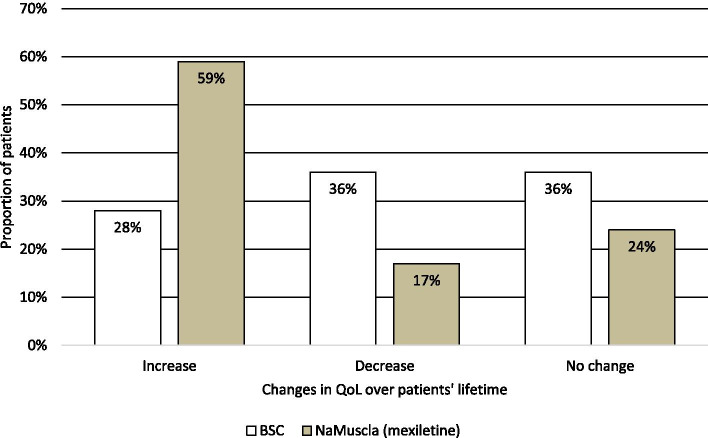
Proportion of adult patients with NDM who experience a change in QoL over their lifetime. Key: BSC, best supportive care

The results of RD1 suggested that for BSC, on average 72% of the patients would be expected to have no change or a decrease in their QoL over their lifetime, half of which are expected to experience a decrease. By contrast, only 41% of patients receiving mexiletine (NaMuscla) would be expected to experience no change or a decrease, of whom only 17% would experience a decrease. In addition, 59% of the patients who are treated with mexiletine (NaMuscla) are expected to experience a disease related QoL increase, while only 28% of the patients receiving BSC are expected to experience a QoL increase (Fig. 3). For those patients who are expected to experience a QoL decline, no consensus was achieved in RD1 on the rate of this decline. However, the majority (67%, 6/9) expected that the QoL of patients receiving BSC would decrease at a faster rate than those receiving mexiletine (NaMuscla). Only a minority of the experts (22%, 2/9) expected the annual rate of decline to be the same; one expert dropped out and therefore did not complete this question (11%, 1/9).

When following up on this in RD2, there was consensus (75%; 6/8) that, on average, the proportion of patients who experience a QoL increase over their lifetime would be higher for those who are treated with mexiletine (NaMuscla) than for patients receiving BSC. Experts were asked to quantify this rate, by selecting one of six answer options ranging from 0% (i.e. no difference in the annual rate at which QoL declines) to 25% (i.e. the annual rate at which QoL declines for patients receiving BSC is 25% faster than for patients treated with mexiletine (NaMuscla)). The outcome of this question suggested that the mean annual rate at which QoL decreases for patients receiving BSC is expected to be 3.7% faster than the corresponding rate for patients treated with mexiletine (NaMuscla) (*n* = 7; median: 3.5%; range: 0–10%).

### Caregiver quality of life

The final section of the Delphi panel study aimed to investigate caregiver QoL in four main aspects (physical health, emotional wellbeing, ability to work or go to school, and the ability to maintain relationships) and assess if there are any perceived differences between caregivers of patients who are treated with mexiletine (NaMuscla) versus those who receive BSC. We did not intend to achieve a consensus on this question, but rather wanted to increase our understanding of the potential impact of NDM on caregiver QoL.

**Fig. 4:**
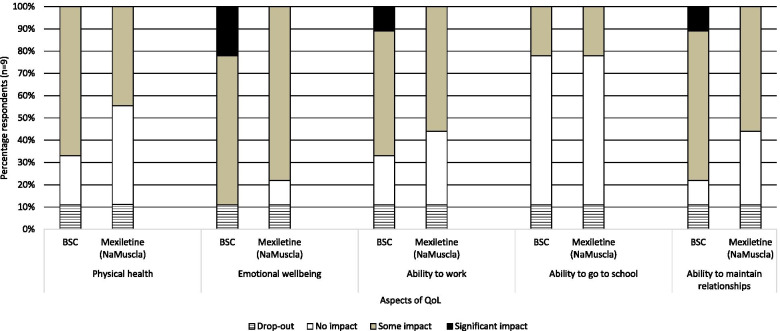
Perceived impact of treatment on aspects of caregiver QoL. Key: QoL, quality of life

The results of this section are presented in Fig. 4. While the opinions of the experts varied, a subtle trend could be observed which suggested a potential positive impact of mexiletine (NaMuscla) treatment on caregiver QoL. In particular, the experts’ responses shifted from expecting some- to a significant impact on aspects of caregiver QoL if the patient receives BSC, to expecting no- to some impact if the patient would receive mexiletine (NaMuscla). Most notably, none of the experts believed that caregivers would experience a significant impact on aspects of their QoL if the patient would be treated with mexiletine (NaMuscla). When a symptomatic patient with NDM would only receive BSC, some experts were of the opinion that caregivers may experience a significant impact on their emotion wellbeing (22%, 2/9), ability to work (11%, 1/9) and ability to maintain relationships (11%, 1/9). While these differences are subtle, they could be of interest to explore in more detail in future studies.

Anecdotes from the experts reflected that the severity of NDM varies and consequently, so does the need for emotional and/or physical support from the caregiver:

‘*Some impact is only seen in caregivers to patients with very severe disabling myotonia. In these cases, caregivers may cut down on work hours and may be psychologically affected’ (C4)*

*‘It will depend on degree and amount of support required. Most NDM patients are not severely disabled but a small minority are. There is a significant impact on shared activities but most NDM patients will be able to be left to care for themselves for most of the time. Physical dependence is likely to be less than psychological dependence on others.’ (C3)*

‘*The physical health, emotional wellbeing and ability to maintain relations of a caregiver will all be impacted due to the additional amount of time that they have to [allocate] to the person [with NDM] that they’re caring for. Chores are not necessarily split evenly, and the carer feels guilty for being unable to maintain relationships with other people’ (C6)*

Three of the seven experts who completed RD2 provided comments related to the impact on the QoL of caregivers of symptomatic patients with NDM who receive mexiletine (NaMuscla) treatment. The responses suggested that the QoL of the caregiver is, unsurprisingly, related to the symptoms of the patient. As mexiletine (NaMuscla) reduces symptoms, it is also expected that fewer caregivers are impacted (i.e. a smaller proportion of caregivers would be impacted) (*n* = 1) or are impacted to a lesser extend (*n* = 1), although some support might still be needed due to residual symptoms (*n* = 2):

*‘[the QoL of caregivers of patients with NDM who receive mexiletine (NaMuscla) is] likely to be improved but some care and support still needed’ (C2)*

*‘fewer caregivers likely would be involved’ (C4)*

*‘Regardless of symptom relief I imagine that there will be some residual symptoms but also that even if symptom free there may be a burden being in a relationship with anyone who has a long-term condition on long-term treatment. I expect this would be more psychological and emotional’.* (C5)

## Discussion

While evidence on the clinical genotype, phenotype and treatment of NDM continues to grow, these studies often include small patient numbers and have not yet addressed wider questions that are crucial for payer and clinical decision making to ultimately improve patient access to treatments. These questions include, for example, how the HRU of patients with NDM who receive mexiletine (NaMuscla) compares to that of patients receiving BSC; how the disease progresses over time in terms of patient QoL; the clinical meaningfulness of trial outcomes; and the real-life dosage of mexiletine (NaMuscla). With this study we aimed to provide a starting point to address these data gaps and explore the viewpoints of leading clinical experts on areas of NDM that are relatively understudied.

For example, we obtained mean values of resource use based on expert opinion which has, to our knowledge, not been studied and published before. While this information is focused primarily on the UK, it provides a needed starting point to estimate the costs associated with NDM and more specifically supports future calculations on the potential cost effectiveness and budget impact of mexiletine (NaMuscla) compared with BSC. The results also highlighted the importance of mental health support, alongside the management of physical complaints, for patients to be able to cope with their symptoms. Estimates from our expert panel suggest that the mean proportion of patients that utilize mental health support is nearly equal to the proportion of patients that require physiotherapy. Consensus was reached that this utilization would be lower for patients who are treated with mexiletine (NaMuscla) compared to those receiving BSC. This seems to underline the importance of considering mental health support as part of the care of NDM and the potential positive impact of treatment with mexiletine (NaMuscla) on the mental health of patients with NDM.

Other results of this Delphi panel study provide context to key outcomes of the MYOMEX trial and supported their relevance to the clinical setting in which NDM experts practise and their patients. For example, our expert panel believed that the improvement in QoL, as measured using the stiffness VAS score and INQoL in the MYOMEX trial, is considered a clinically meaningful difference. Moreover, the experience of our clinical panel suggests that the average daily dose of mexiletine (NaMuscla) in the long-term is expected to be two capsules a day, which provides valuable insight on how this product can be successfully used outside the confines of the RCT restrictions.

We explored the possibility of mapping domains of the INQoL to the EQ-5D based on their similarity, to supplement a study from Lloyd et al. (2021, data on file), which aimed to derive utility estimates from the INQoL. Our findings suggest that domains of the INQoL and the EQ-5D have similarities from a clinical perspective, particularly for the ‘pain’, ‘usual activities’ and ‘anxiety/depression’ domains. While we acknowledge the limitations of conducting such an exercise using a Delphi panel, the findings are in line with the results from Lloyd et al. (2021, data on file) and support the idea of conceptual overlap between the two QoL instruments and, therefore, the possibility of mapping outcomes of one to the other.

What is perhaps even more interesting is the anecdotal evidence from this study which provides additional insight into the perceived impact on QoL of NDM on the patient as well as the caregiver, including disease progression. Expert opinion suggested that the QoL of patients with NDM might not be static and may change over time, both for the positive as well as the negative. However, our expert panel expected that a larger proportion of patients with NDM would experience a QoL improvement over their lifetime if they receive treatment with mexiletine (NaMuscla), compared to patients who only receive BSC. In addition, for patients whose QoL decreases, it was expected that this would decline at a slower rate for patients treated with mexiletine (NaMuscla) compared to patients who only received BSC. However, there was uncertainty about the rate of this decline.

Previous studies have shown that the QoL of not only patients with neuromuscular disease, but also of their caregivers, can be negatively impacted [41, 42]. While literature around caregiver QoL in NDM is lacking, our results suggest that clinicians indeed believe that caregivers are to some degree impacted in some domains of their QoL, particularly their emotional wellbeing. This is potentially more marked when a symptomatic patient only receives BSC. Because NDM affects patients from a young age, we anticipate this impact to be even more pronounced for caregivers of paediatric patients.

Several limitations of this study should be highlighted. As with any Delphi panel, the robustness and generalizability of the results are linked to the number of experts, the degree of their expertise and the number of rounds. While the expert numbers were small in this study, they were within the recommended range for this type of study. Guidance has suggested that the optimum size of a panel is between seven and 12 members [43]. However, it should be noted that our panel consisted of clinical experts, it could be interesting to conduct a similar study among patient experts and informal caregivers to get more insight in the patient perspective. In addition, a Delphi panel does not allow for open communication between experts and the moderators. We believe that this made it more challenging for the experts to consider the more conceptual elements of this exercise, such as the mapping of the INQoL to the EQ-5D. Finally, our study presents the viewpoints of experts on two main management strategies: treatment with mexiletine (NaMuscla), which is considered the preferred first-line myotonia treatment option and is the only licensed product in the EU for NDM, and BSC. In real-life, patients may be treated with other (off-label) treatment options for myotonia. Moreover, given the objectives of our study, we did not investigate treatment pathways and the influence of switching treatments for myotonia. To get a more holistic view of the cost associated with NDM and the benefits of other off label treatments in comparison to mexiletine, future studies are needed to explore this in more detail. For example, a randomised phase III non-inferiority study (MEND) is currently in development to compare lamotrigine with mexiletine, outcomes of this study may shed some light on part of these gaps [44].

This study was conducted during the first wave of the COVID-19 pandemic. Several panellists indicated they were under immense pressures, which made it more challenging to commit to this study. We feel that these exceptional circumstances may have led to the experts dropping out in RD1 and RD2 and subsequently made achieving consensus (especially around HRU) more difficult.

## Conclusion

In the field of rare diseases, such as NDM, decision makers often have to make decisions about the reimbursement of treatments under time pressure and based on limited (clinical) evidence and/or evidence based on small patient numbers. This study successfully provided additional information that is required for health economic modelling and to support reimbursement decision makers. In addition, the results may support clinicians’ decision on treatment of NDM patients. We believe that the findings from this Delphi panel provide more confidence to payer and clinical decision makers by reducing some of the areas of uncertainty and that it will provide a starting point for future research in this area, to ultimately ensure that patients with NDM can access effective treatments in a more timely way.

## Supplementary Information

**Additional file 1.**

**Additional file 2.**

**Additional file 3.**

## Data Availability

The datasets generated and/or analysed during the current study are not publicly available due to the low number of participants, to protect their anonymity. When available, aggregated level data are available from the corresponding author on reasonable request (mschurer@bresmed.com).

## Abbreviations

BSC: Best supportive care
HTA: Health technology assessment
INQoL: The Individualized Neuromuscular Quality of Life Questionnaire
EQ-5D: The EuroQol five-dimension questionnaire
MYOMEX: The Efficacy and Safety of Mexiletine (NaMuscla) in Non-Dystrophic Myotonias trial
NDM: Non-dystrophic myotonia
QoL: Quality of life
VAS: Visual analogue scale
