# Supplementary material for: Improving the understanding of how patients with non-dystrophic myotonia are selected for myotonia treatment with mexiletine (NaMuscla): outcomes of treatment impact using a European Delphi panel
Source: BMC Neurol. 2021 Dec 1;21:467. doi: 10.1186/s12883-021-02491-3 (PMC8633892; doi:10.1186/s12883-021-02491-3)
Supplement: Supplementary file 1 — Additional file 1. [file 12883_2021_2491_MOESM1_ESM.pdf]

Title: Improving the understanding of how patients with non-dystrophic myotonia are selected for myotonia treatment with mexiletine (NaMuscla): outcomes of treatment impact using a European Delphi panel

Authors: Ann-Marie Chapman[1], Marieke Schurer[2*], Laure Weijers[2], Amer Omar[3], Hiba Lee[4], Alla Zozulya-Weidenfeller[3], Crispin Ellis[5], Shaneil Sonecha[5], Christiane Schneider-Gold[6]

***Correspondence:** mschurer@bresmed.com

[1] BresMed Health Solutions Ltd, Sheffield, UK

[2] BresMed Netherlands BV, Utrecht, The Netherlands

[3] Lupin Atlantis Holdings SA, Zug Switzerland

[4] AGH Partners Ltd, London, UK

[5] Lupin Healthcare Ltd, Slough, UK

[6] St. Josef-Hospital, Bochum, Germany

Delphi panel to support the submission of NaMuscla for the treatment of non-dystrophic myotonia, round 1 survey

Background

On behalf of Lupin Healthcare Ltd and BresMed Health Solutions Ltd, thank you for agreeing to participate in this Delphi Panel research project. The project focuses on non-dystrophic myotonia (NDM) in the UK setting primarily to support refinement of a cost-effectiveness model.

The Delphi methodology

The Delphi methodology was originally developed by the RAND Corporation in the 1950s as a practical and structured method of obtaining opinions on a given question from a range of experts.[1] The participants take part anonymously in sequential rounds of surveys, with each round being refined based on the feedback from the previous version. The goal is to reach a consensus on the questions posed. This project will comprise two rounds of surveys, with each round taking no more than 1 hour. A synthesis of responses will be conducted between each survey round to formulate the subsequent surveys.

As per the Delphi Process, your responses will remain anonymous to the other Delphi Panel respondents. The final report will acknowledge your participation although no statements or responses attributable to you or others will be included in this report. This aspect of the Delphi process is designed to prevent dominance of individual opinions, thus enabling the most robust possible consensus.[2]

Cost-effectiveness models

The objective of a cost-effectiveness model is to assess whether an intervention is an efficient use of National Health Service (NHS) resources[3], which can be used to support decision making in the health technology assessment context. Within the UK, results of such a model are a requirement of the Scottish Medicines Consortium (SMC). In addition, the National Institute for Health and Care Excellence (NICE) requires the submission of an economic model. This Delphi Panel intends to support the refinement of a cost-effectiveness model and submissions to NICE, the SMC and potentially other European markets for NaMuscla® (mexiletine) for patients with NDM by addressing pre-identified data gaps and areas of uncertainty.

Gaps and uncertainties have been identified on healthcare utilization, natural history of the condition, instruments to measure quality of life (QoL) and caregiver QoL. Given the above, the research objectives of this Delphi Panel are as follows:

Number	Objective
1	To investigate the healthcare utilization of patients with NDM who are on treatment with NaMuscla (mexiletine) compared to patients who receive BSC, from the perspective of the UK NHS and Personal Social Services
2	Explore how useful the INQoL is and identify which attributes of INQoL reflect the domains of the EQ-5D best
3	Understand how NDM may progress over time, in terms of patients' QoL
4	Estimate the impact of NDM on caregivers' QoL
**Key**: BSC, best supportive care; EQ-5D, EuroQoL 5D questionnaire; INQoL, Individualized Neuromuscular Quality of Life Questionnaire; NDM, non-dystrophic myotonia; NHS, National Health Service; QoL, quality of life.	

Delphi panel to support the submission of NaMuscla for the treatment of non-dystrophic myotonia, round 1 survey

Round 1 survey

This is the first round of two surveys and should take approximately 1 hour to complete. We recommend that you complete the survey in one sitting; however, if this is not possible, you can save your answers and complete the survey at a more convenient time. For answers to be saved, you will need to complete the section you are on and press 'Next'.

Your individual responses to this survey will be kept anonymous and will be analysed by BresMed. Results will be combined and presented back to you in a second survey with the aim of moving towards a consensus.

Delphi panel to support the submission of NaMuscla for the treatment of non-dystrophic myotonia, round 1 survey

Adverse event reporting

Although this is an online survey and how you respond will be treated in confidence, should you raise an adverse event and/or product complaint, we will need to report this, even if it has already been reported by you directly to the company or the regulatory authorities using the Medicines and Healthcare products Regulatory Agency's 'Yellow Card' system, or in line to respective national reporting schemes as outlined on http://www.adrreports.eu/.

If any adverse events are identified during the analysis of responses, we require your permission to include your name and contact information in the report we send to the pharmaceutical company commissioning this market research, so that they can report this and meet their legal obligations. The drug safety department may wish to contact you directly for further information relating directly to the adverse event. Everything else you contribute during the Delphi survey will continue to remain confidential.

* 1. Are you happy to proceed with the survey on this basis?

◯ Yes

◯ No

Delphi panel to support the submission of NaMuscla for the treatment of non-dystrophic myotonia, round 1 survey

About you

* 2. What is your name?
Please note that this information will be used for internal tracking purposes only. Your individual responses will remain anonymous.

* 3. I am a

- Neurologist
- Specialist nurse
- Other, please specify:

* 4. Do you have experience using mexiletine for the treatment of NDM?

* 5. How many years of experience do you have in managing patients with NDM?

* 6. How many patients with NDM are currently registered in the centre/hospital where you work?

* 7. Approximately how many patients with NDM are currently under your care?

* 8. In which country do you live and work?

The next section of the Delphi panel will be about healthcare resource utilization (HRU) associated with NDM. Please note that for experts from outside the UK, we cannot anonymize your answers as only one expert per non-UK country is participating.

9. Are you happy to continue to the HRU section?

- ◯ Yes, I am happy for my non-anonymized answers on HRU to be used in the Delphi
- ◯ No, I would like to skip the questions related to HRU

Delphi panel to support the submission of NaMuscla for the treatment of non-dystrophic myotonia, round 1 survey

Healthcare resource utilization

We would like to know what healthcare resources are utilized by patients who are on best supportive care (BSC) compared to patients who are on treatment with NaMuscla (mexiletine), from the perspective of your country's National Health Service. For the purpose of this survey, BSC is defined as any supportive care that symptomatic adult patients with NDM may receive that does not involve symptom-modifying pharmacological treatment. However, BSC does include for example, the use of supportive medication (e.g. pain killers), mobility aids, physiotherapy or speech therapy.

Please note: It is assumed that all patients, including those receiving BSC as well as those treated with NaMuscla (mexiletine), require initial diagnostic testing. Therefore, diagnostic tests are not considered in the below questions.

Table 1 shows a simple estimation of the percentage of patients that will need each type of resource in a year, as used in the UK economic model. The estimated percentages are assumptions informed by a combination of patient and clinical expert elicitation interviews and an expert advisory board from the UK.

Questions 10 and 11 will ask about the percentage of patients that utilize each type of resource per year, based on your own experience. We will ask about the number of units that are used by patients who use each resource in questions 13 and 14.

Table 1: Estimated percentages of patients with NDM that require each type of health resource used in the UK economic model. Estimates are based on UK expert elicitation and an ad board.

Health resource	NaMuscla (mexiletine)	BSC
Physiotherapy	18%	45.6%
Occupational therapist*	16%	20.8%
Speech therapy	12%	21.6%
Day case attendances**	100%	100%
Use of wheelchair	0%	0%
Use of walking stick	0.8%	8.8%
Use of walking frame	0.4%	4.4%
No mobility aid	99%	87%
Hospital admission for fracture	10%	20%

Key: BSC, best supportive care.

Notes: *Occupational therapy is a healthcare profession that focuses on developing, recovering, or maintaining the daily living and working skills of people with physical, mental, or cognitive impairments. Outside the UK this may also be referred to as ergotherapy[4]; **Day case attendances include consultant led multi-professional, non-admitted, face-to-face attendance and follow-up. A consultant physician is a senior doctor who practises in one of the medical specialties[5]

Explanation table: a value of 18% in the NaMuscla (mexiletine) column for physiotherapy means that it is expected that 18% of the NDM patients treated with NaMuscla (mexiletine) will require physiotherapy in a year.

* 10. From your experience, please provide an estimation of the percentage of adult patients with NDM, who receive **BSC**, that would use each type of resource in the table below. Please ensure that the values are between 0-100%.

Physiotherapy

Occupational therapist

Speech therapy

Day case attendances

Use of wheelchair

Use of walking stick

Use of walking frame

Hospital admission for fracture

* 11. From your experience, please provide an estimation of the percentage of adult patients with NDM, who are treated with **NaMuscla (mexiletine)**, that would use each type of resource in the table below. Please ensure that the values are between 0-100%.

Physiotherapy	
Occupational therapist	
Speech therapy	
Day case attendances	
Use of wheelchair	
Use of walking stick	
Use of walking frame	
Hospital admission for fracture	

12. If there are any other items of resource use in secondary and other care settings that are missing from the table above, please list them here:

Resources in secondary care setting	
Resources in other care settings	

Table 2 shows the annual **number of units** required per patient with NDM who use a health resource, as used in the UK economic model. The estimated number of units used per patient in the NaMuscla (mexiletine) group is based on an expert advisory board with experts from Scotland and England. For the group who receives BSC, assumptions have been made

Table 2: Annual number of healthcare resource units required for those treated with mexiletine versus best supportive care used in the UK economic model. Estimates are based on an advisory board with UK experts

Health resource	NaMuscla (mexiletine)	BSC
Physiotherapy	6	18
Occupational therapist*	6	18
Speech therapy	6	18
Day case attendances**	1	3.96

Key: BSC, best supportive care.

Notes: *Occupational therapy is a healthcare profession that focuses on developing, recovering, or maintaining the daily living and working skills of people with physical, mental, or cognitive impairments. Outside the UK this may also be referred to as ergotherapy[4]; **Day case attendances include consultant led multi-professional, non-admitted, face-to-face attendance, and follow-up. A consultant physician is a senior doctor who practises in one of the medical specialties.[5]

Explanation table: a value of six in the NaMuscla (mexiletine) column for physiotherapy means that it is expected that of those treated with NaMuscla (mexiletine) who require physiotherapy, on average each patient will require six physiotherapy visits in a year.

* 13. Of the adult patients with NDM who receive **BSC** who make use of a resource, please provide an estimation of how often that patient would use the resource per year (in numbers).

Physiotherapy

Occupational therapist

Speech therapy

Day case attendances

* 14. Of the adult patients with NDM treated with **NaMuscla (mexiletine)** who make use of a resource, please provide an estimation of how often that patient would use the resource per year (in numbers).

Physiotherapy

Occupational therapist

Speech therapy

Day case attendances

* 15. It has been noted from clinician insights that patients with NDM may require mental health support due to their myotonic symptoms.

Considering your patients with NDM who are treated with BSC or NaMuscla (mexiletine), what proportion of patients would require (any form of) mental health support? Please provide your answer in percentages (0-100%):

Proportion of patients treated with **BSC** requiring mental health support:

Proportion of patients treated with **NaMuscla (mexiletine)** requiring mental health support:

* 16. From your experience, what mental health care resources would patients with NDM use?

* 17. **If you are an expert from outside the UK**, could you please provide an estimate of how long each of the below visits will take on average (in minutes)?
If you are an expert from the UK, please note 'UK' in the text boxes.

Physiotherapy

Occupational therapist

Speech therapy

Day case attendances

Treatment with NaMuscla (mexiletine)

The Summary of Product Characteristics of NaMuscla (mexiletine) states that the recommended starting dose is 167 mg daily (i.e. one capsule daily, or 200 mg of mexiletine hydrochloride). Based on clinical response, the dose can be increased to 333 mg daily (i.e. 2 capsules daily, or 400 mg of mexiletine hydrochloride) after at least one week of treatment. After at least another further week, this dose can be increased to a maximum of 500 mg daily (i.e. 3 capsules daily, or 600 mg of mexiletine hydrochloride) based on clinical response. Maintenance treatment is between 167–500 mg daily (200mg to 600mg mexiletine hydrochloride daily), which equates to 1–3 capsules per day, according to the intensity of symptoms and clinical response, taken throughout the day.[6]

Suetterlin et al. (2015)[7] (please see the pre-reading material) investigated the long-term safety and efficacy of mexiletine in adult patients with NDM in a retrospective cohort study. A total of 63 patients were included in the study with a mean length of follow-up of 4.8 years (range 6 months to 17.8 years). Overall, they found the real life long-term mean effective daily dose varied between 333 mg–550 mg of mexiletine hydrochloride, depending on the genotype (please see Table 3). The mean of all of the genotypes was 417mg mexiletine hydrochloride (or 2.08 capsules) daily.

Table 3: Mexiletine mean effective dose by genotype.

	SCN4A Missense	*CLCN1* Missense	*ClCN1* Hom NMD	*CLCN1* Het NMD
Effective dose of mexiletine hydrochloride mean (SD) [No.], mg	333 (177) [21]	550 (85) [10]	463 (160) [8]	420 (175) [10]
Source: Suetterlin et al. 2015.[7]				

In the next question we would like to explore how many capsules of NaMuscla (mexiletine) you expect an adult patient to take in real life on average per day. Please note that this may deviate from what you would prescribe the patient.

* 18. Considering an adult patient with NDM who receives NaMuscla (mexiletine) in **real life**, on average how many capsules would you expect a patient to take per day **in the long term**?

- One capsule per day (i.e. 167 mg NaMuscla (mexiletine)/200 mg mexiletine hydrochloride daily)
- Two capsules per day (i.e. 333 mg NaMuscla (mexiletine)/400 mg mexiletine hydrochloride daily)
- Three capsules per day (i.e. 500 mg NaMuscla (mexiletine)/600 mg mexiletine hydrochloride daily)
- Other, please specify:

* 19. Please tick all boxes that apply. I would consider adult patients with NDM eligible for NaMuscla (mexiletine) treatment if they:

- [ ] Have genetically confirmed NDM
- [ ] Have symptoms severe enough to treat with NaMuscla (mexiletine) which impact their daily lives
- [ ] Are any age over 18
- [ ] Are drug naïve or those receiving NaMuscla (mexiletine)/ or other off-licensed treatments
- [ ] Have a normal cardiac exam as performed by a cardiologist, including EKG and cardiac ultrasound
- [ ] Can be treated subject to NaMuscla (mexiletine) being available
- [ ] Can be treated subject to NaMuscla (mexiletine) being approved by the funder for reimbursement
- [ ] Other, please specify:

Instruments to measure quality of life and disease severity

When answering the below questions, please carefully review the QoL instruments that were sent in the pre-reading materials (i.e. the Individualized Neuromuscular Quality of Life Questionnaire v1.2 [INQoL], the EQ-5D, and the Visual Analogue Scale [VAS]).

The Efficacy and Safety of Mexiletine in Non-dystrophic myotonias (MYOMEX) trial, was a multi-centre, double-blind, placebo-controlled cross-over study to compare the effects of mexiletine versus placebo in patients with myotonia congenita and paramyotonia congenita.

In the MYOMEX trial, the VAS outcomes were measured as one of the primary outcomes of efficacy. The VAS is a scale which was used by patients to indicate how much stiffness they experience, ranging from 0 (no stiffness) to 100 (worst possible stiffness).

As a secondary outcome of efficacy, the INQoL was used to measure changes in overall QoL. The score of INQoL ranges from 0–100, with higher scores indicating poorer QoL.

The results of the MYOMEX trial for VAS and INQoL are shown in Table 4 and Figures 1-3.

Table 4: Results for the primary and secondary QoL outcomes in the modified intention to treat population of MYOMEX.

Outcome	Mexiletine (n=25)	Placebo (n=25)
**VAS stiffness score**		
Median (range) stiffness VAS score at baseline	71.0 (11, 100)	81.0 (27, 98)
Median stiffness VAS score at end of treatment	16.0	78.0
**Absolute change in median (range) stiffness VAS score from baseline**	**-42.0 (-93, 35)**	**+2.0 (-94, 35)**
Percentage change in stiffness VAS score from baseline	-78%	+2%
Percentage of patients with an absolute change in stiffness VAS from baseline ≥50 mm at end of treatment	57% (12/21)	14% (3/22)
**INQoL**		
Mean (SD) INQL – overall quality of life at baseline	47.8 (20.4)	
**Absolute mean (SD) change in INQL from baseline**	**-20.7 (24.6)**	**2.6 (15.0)**

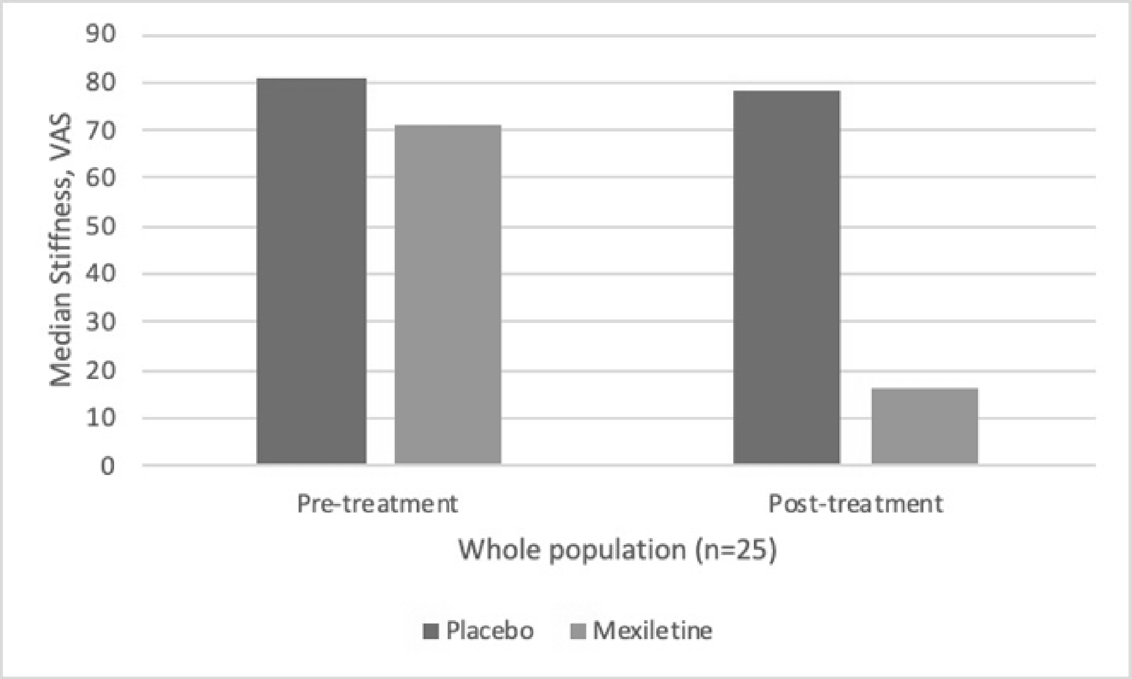

Figure 1: Median evolution of stiffness using the visual analogue score
Treatment effect (p< 0.001)

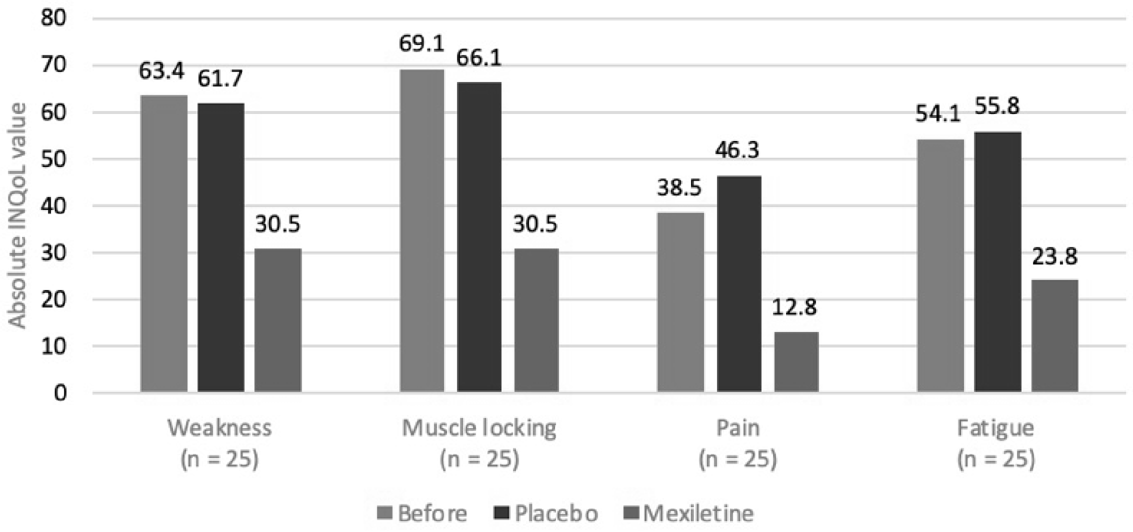

Figure 2: Scores for INQoL symptom subdomains before study initiation and in treatment and no treatment arms of study (mITT)
Treatment effect for each domain of the INQoL questionnaire (p< 0.001)

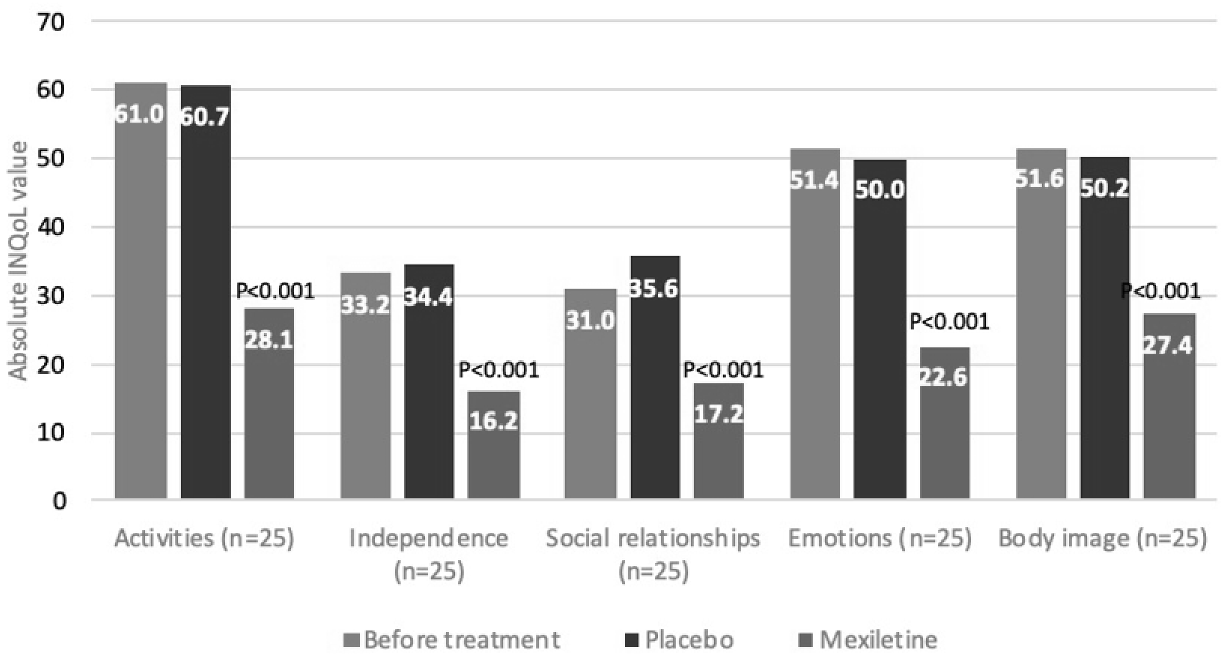

Figure 3: Scores for INQoL impact of daily living domains before study initiation and in treatment and no treatment arms of study (mITT)

* 20. When looking at the absolute change in median stiffness VAS score in the mexiletine arm from baseline, would you consider this to be a clinically important difference?

- Yes
- No
- I am unsure

Please explain your answer:

* 21. When looking at the absolute mean change in the overall QoL score of the INQoL in the mexiletine arm from baseline, would you consider this to be a clinically important difference?

- Yes
- No
- I am unsure

Please explain your answer:

Matching exercise: matching domains of INQoL to domains of the EQ-5D

The INQoL v1.2 consists of 45 questions (or items) within 10 sections (or domains). Four domains measure the impact of common muscle disease symptoms (weakness, locking (aka myotonia), pain and fatigue). Five domains measure the influence of the muscle disease on particular areas of life (activities, independence, relationships, emotions and body image). The last section is related to disease treatment. Please note that the section/ domain on “treatment” is not included in the overall INQoL scoring, and therefore not relevant for this mapping exercise.

In the following question we will ask you to match domains of the INQoL to domains of the EQ-5D which you believe have the greatest conceptual similarity. To be able to complete this question, please ensure you have the EQ-5D and INQoL in front of you.

Example: matching exercise domains: If you think that the ‘mobility’ domain of the EQ-5D is best reflected by domain 1 (‘Your muscle weakness’) of the INQoL, you would tick the box of ‘your muscle weakness’ in the table of the next question.

* 22. Please complete the table below by indicating which domain(s) of the INQoL you believe best match each domain of the EQ-5D, by ticking the relevant boxes.

- You can match multiple domains of the INQoL to a domain of the EQ-5D if you wish.
- However, please match each unique domain of the INQoL only once (e.g. match it to only one domain of the EQ-5D).
- **This means that you can provide multiple answers per row but only one answer per column**

Please note, after a pilot with an independent expert and subsequent internal discussions, it was found that 'the things you do' (activities) section required further subdivision, as per the table provided.

	Your muscle weakness	The locking of your muscles	Your pain	How tired you feel/ Fatigue	The things you do - daily activities	The things you do – leisure and work activities	Your independence	Your Relationships	How you feel/ emotions	The way you look/ Body Image
Mobility	☐	☐	☐	☐	☐	☐	☐	☐	☐	☐
Self-care (washing & dressing)	☐	☐	☐	☐	☐	☐	☐	☐	☐	☐
Usual activities (leisure, work and social activities)	☐	☐	☐	☐	☐	☐	☐	☐	☐	☐
Pain/ discomfort	☐	☐	☐	☐	☐	☐	☐	☐	☐	☐
Anxiety/ depression	☐	☐	☐	☐	☐	☐	☐	☐	☐	☐

If you have any comments you would like to share on this matching exercise, please list them here:

Matching exercise: matching items of INQoL to domains of the EQ-5D

Now that you have matched domains of INQoL to domains of EQ-5D, we would like you to review the questions (or **items**) within each of the domains you listed in Question 15. We will ask you to indicate what items of the INQoL you believe best match the domains of EQ-5D.

Example matching exercise items: This example assumes that someone previously answered that the mobility domain of the EQ-5D is most similar to Domain 1 of the INQoL (see also the previous example).

If you think that item 1a ('How much weakness would you say you have in the muscles affected by your condition?') of the INQoL matches best to the 'mobility' domain of the EQ-5D, you would list this as follows:

Domain of EQ-5D	Matching item(s) of INQoL
Mobility	1a

* 23. Based on your answer to the previous question, please complete the table below by indicating which item(s) of the INQoL you believe best match each domain of the EQ-5D.

- You can match multiple items of the INQoL to one domain of the EQ-5D if you wish
- However, please match a unique item of the INQoL only once (e.g. match it to only one domain of the EQ-5D).
- **For example, this means that if you have already matched item 1a to the mobility domain, please do not match it to any other domain of EQ-5D (similar to the previous question).**

Mobility	
Self-care (washing & dressing)	
Usual activities (leisure, work and social activities)	
Pain/discomfort	
Anxiety/depression	

24. If you have any comments you would like to share on this matching exercise, please list them here:

* 25. In your opinion, what domains of the INQoL v1.2 influence the QoL for patients with NDM the most. Please rank the domains from 1 (most impactful driver of QoL) to 10 (least impactful driver of QoL)

Your muscle weakness

The locking of your muscles

Your pain

How tired you feel/ Fatigue

The things you do - Daily activities

The things you do - Leisure and work activities

Your independence

Your Relationships

How you feel/ emotions

The way you look/ body image

Natural history

Much is still unknown about the natural history and determinants of morbidity in NDM.[8] However, several studies suggest that QoL worsens over time in the absence of treatment. In a Dutch cross-sectional study, Trip et al.[9] found that 58% (n=36) of the patients reported that the severity of their myotonia had increased since the onset of their symptoms. Similarly, findings from previous surveys from Janet Stone (2019) amongst UK patients with NDM (see Figure 4)[10] showed that 87.3% (n=21) and 70.8% (n=17) of the patients experienced a worsening of their stiffness and weakness since diagnosis, respectively. Feedback from clinical experts and patient interviews conducted by Lupin also suggested that QoL decreases over time without treatment.

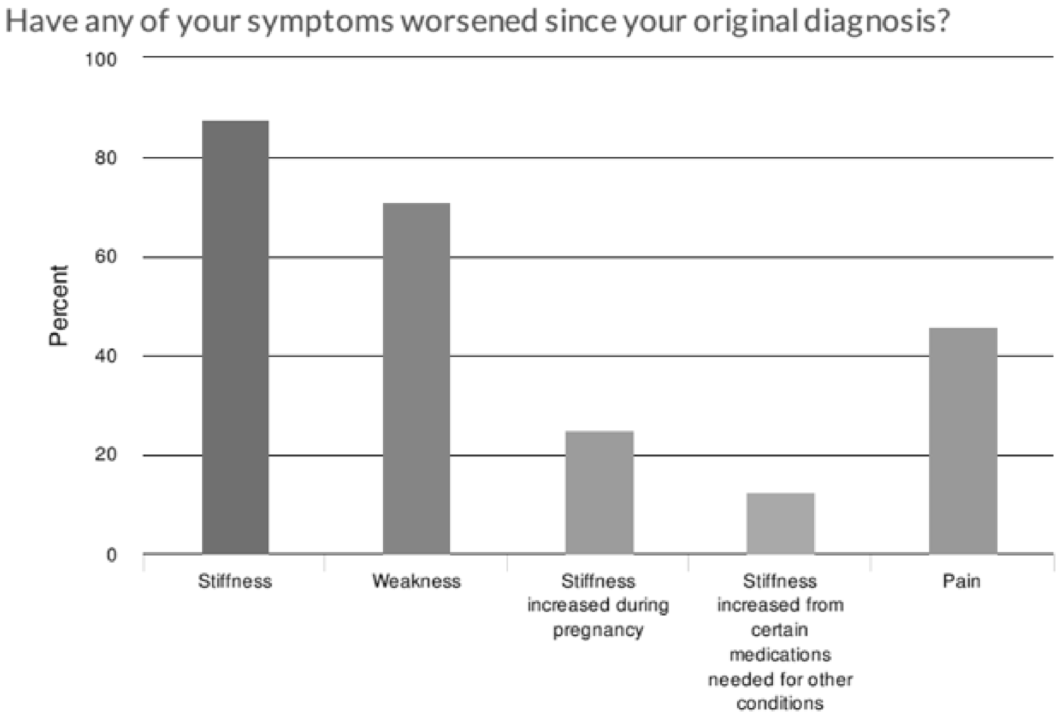

Figure 4: Percentage of patients that experienced a worsening of their symptoms since diagnosis
Results of a survey conducted by Janet Stone[10]

In the following questions we would like to explore this topic in more detail and investigate if there are any differences in the progression of QoL between patients who receive BSC, compared to those who receive NaMuscla (mexiletine) on a regular basis.

* 26. From your experience, please indicate what proportion of adult patients with NDM who receive **BSC** will experience an overall increase, decrease or no change in their disease-related QoL over their lifetime. Please provide your answer in percentages (0-100) and ensure the total adds up to 100%.

Increase

Decrease

No change

* 27. From your experience, please indicate what proportion of adult patients with NDM who are treated with **NaMuscla (mexiletine)** will experience an overall increase, decrease or no change in their disease-related QoL over their lifetime. Please provide your answer in percentages (0-100) and ensure the total adds up to 100%.

Increase

Decrease

No change

* 28. For those patients who experience a decline in their QoL, do you expect the **annual rate** at which disease-related QoL decreases over time will be different for patients who receive BSC compared to patients treated with NaMuscla (mexiletine)?

- No, I expect that the annual rate at which QoL decreases will be the same
- Yes, I expect that the QoL of patients receiving BSC will decrease at a faster rate annually compared to patients treated with NaMuscla (mexiletine)
- Yes, I expect that the QoL of patients receiving BSC will decrease at a slower rate annually compared to patients treated with NaMuscla (mexiletine)

Please explain your answer:

Delphi panel to support the submission of NaMuscla for the treatment of non-dystrophic myotonia, round 1 survey

Caregiver quality of life

Considering the symptoms of NDM it is expected that NDM will have some impact on the QoL of families and caregivers of patients.

* 29. Considering symptomatic adult patients with NDM **who receive BSC**, please indicate the impact on QoL you might expect for a primary caregiver (e.g. their spouse).

	No impact	Some impact	Significant impact
Physical health	○	○	○
Emotional wellbeing	○	○	○
Ability to work	○	○	○
Ability to go to school	○	○	○
Ability to maintain relationships	○	○	○

For the answers in which you indicated that there is some or significant impact, please specify how you would expect these to be impacted (e.g. a reduction in working hours to be able to care for the patient, caregiver spending more time on household activities to support the patient, caregiver feeling isolated or anxious).

* 30. Considering symptomatic adult patients with NDM **who receive treatment with Namuscla (mexiletine) on a regular basis**, please indicate the impact on QoL you expect for their primary caregiver (e.g. their spouse).

	No impact	Some impact	Significant impact
Physical health	◯	◯	◯
Emotional wellbeing	◯	◯	◯
Ability to work	◯	◯	◯
Ability to go to school	◯	◯	◯
Ability to maintain relationships	◯	◯	◯

For the answers in which you indicated that there is some or significant impact, please specify how you would expect these to be impacted (e.g. a reduction in working hours to be able to care for the patient, caregiver spending more time on household activities to support the patient, caregiver feeling isolated or anxious).

End

This is the end of the survey, thank you for your participation. We will analyse all responses and will contact you again within the next few weeks regarding the second round of the Delphi.

If you have any questions or comments, please do not hesitate to contact Marieke Schurer (Senior insight analyst, BresMed) via: mschurer@bresmed.com. Alternatively, write your comments or questions in the text box below.

A list of references is shown on the next page.

31. If you have any comments on the survey, please write them here:

References

1. **Dalkey N and Helmer O. An experimental application of the Delphi method to the use of experts. Manage Sci. 1963; 9: 458-67.**
2. **Iglesias CP, Thompson A, Rogowski WH and Payne K. Reporting guidelines for the use of expert judgement in model-based economic evaluations. Pharmacoeconomics. 2016; 34: 1161-72.**
3. **National Institute for Health and Care Excellence (NICE). Guide to the methods of technology appraisal. 2013.**
4. **Royal College of Occupational Therapists. www.rcotss-ip.org.uk/what-is-occupational-therapy. Accessed on 13/02/2020.**
5. **Royal College of Physicians. www.rcplondon.ac.uk/education-practice/advice/consultant-physicians. accessed on 13/02/20.**
6. **Electronic medicines compendium. Namuscla 167 mg hard capsules. Summary of product characteristics. 2018.**
7. **Suetterlin KJ, Bugiardinin E, Kaski JP, et al. Long-term safety and efficacy of mexiletine for patients with skeletal muscle channelopathies. JAMA. 2015; 72: 1531-2.**
8. **Matthews E, Fialho D, Tan SV, et al. The non-dystrophic myotonias: molecular pathogenesis, diagnosis and treatment. Brain. 2010; 133: 9-22.**
9. **Trip J, de Vries J, Drost G, Ginjaar HB, van Engelen BG and Faber CG. Health status in non-dystrophic myotonias: close relation with pain and fatigue. Journal of neurology. 2009; 256: 939-47.**
10. **Stone J. NDM survey UK Respondents, (data on file). 2019.**

Delphi panel to support the submission of NaMuscla for the treatment of non-dystrophic myotonia, round 1 survey

End of survey

Thank you for your response.

Please contact Marieke Schurer (mschurer@bresmed.com) to discuss any concerns you may have about this study or our adverse event reporting obligations before continuing the survey.
