# Supplementary material for: Improving the understanding of how patients with non-dystrophic myotonia are selected for myotonia treatment with mexiletine (NaMuscla): outcomes of treatment impact using a European Delphi panel
Source: BMC Neurol. 2021 Dec 1;21:467. doi: 10.1186/s12883-021-02491-3 (PMC8633892; doi:10.1186/s12883-021-02491-3)
Supplement: Supplementary file 2 — Additional file 2. [file 12883_2021_2491_MOESM2_ESM.pdf]

Title: Improving the understanding of how patients with non-dystrophic myotonia are selected for myotonia treatment with mexiletine (NaMuscla): outcomes of treatment impact using a European Delphi panel

Authors: Ann-Marie Chapman[1], Marieke Schurer[2*], Laure Weijers[2], Amer Omar[3], Hiba Lee[4], Alla Zozulya-Weidenfeller[3], Crispin Ellis[5], Shaneil Sonecha[5], Christiane Schneider-Gold[6]

***Correspondence:** mschurer@bresmed.com

[1] BresMed Health Solutions Ltd, Sheffield, UK

[2] BresMed Netherlands BV, Utrecht, The Netherlands

[3] Lupin Atlantis Holdings SA, Zug Switzerland

[4] AGH Partners Ltd, London, UK

[5] Lupin Healthcare Ltd, Slough, UK

[6] St. Josef-Hospital, Bochum, Germany

Delphi panel to support the submission of NaMuscla for the treatment of non-dystrophic myotonia, round 2 survey

Background

On behalf of Lupin Healthcare Ltd and BresMed Health Solutions Ltd, thank you for your continued participation in this Delphi panel research project. The project focuses on non-dystrophic myotonia (NDM) to support the refinement of a cost-effectiveness model. The research objectives of this Delphi panel are shown in Table 1.

Table 1: Research objectives

Number	Objective
1	To investigate the healthcare utilization of patients with NDM who are on treatment with NaMuscla® (mexiletine) compared with patients who receive BSC, from the perspective of the UK NHS and Personal Social Services
2	Explore how useful the INQoL is and identify which attributes of INQoL best reflect the domains of the EQ-5D
3	Understand how NDM may progress over time, in terms of patients' QoL
4	Estimate the impact of NDM on caregivers' QoL
**Key**: BSC, best supportive care; EQ-5D, EuroQol 5D questionnaire; INQoL, Individualized Neuromuscular Quality of Life Questionnaire; NDM, non-dystrophic myotonia; NHS, National Health Service; QoL, quality of life.	

Delphi panel to support the submission of NaMuscla for the treatment of non-dystrophic myotonia, round 2 survey

Round 2 survey

Your responses to the first survey have been collated and reviewed (eight completed surveys were received and analysed). This second round of the Delphi panel presents the analysis and asks additional questions that aim to further explore the topics and move towards a consensus of opinion.

We anticipate that this individual survey should take approximately 1 hour to complete. We recommend that you complete the survey in one sitting; however, if this is not possible, you can save your answers and complete the survey at a more convenient time. To save your answers, you will need to complete the section you are on and press 'Next'.

Your individual responses to this survey will be kept anonymous and will be analysed by BresMed.

Adverse event reporting

Although this is an online survey and how you respond will be treated in confidence, should you raise an adverse event and/or product complaint, we will need to report this, even if it has already been reported by you directly to the company or the regulatory authorities using the Medicines and Healthcare products Regulatory Agency's 'Yellow Card' system, or in line to respective national reporting schemes as outlined on http://www.adrreports.eu/.

If any adverse events are identified during the analysis of responses, we require your permission to include your name and contact information in the report we send to the pharmaceutical company commissioning this market research, so that they can report this and meet their legal obligations. The drug safety department may wish to contact you directly for further information relating directly to the adverse event. Everything else you contribute during the Delphi survey will continue to remain confidential.

* 1. Are you happy to proceed with the survey on this basis?

- ◯ Yes
- ◯ No

Delphi panel to support the submission of NaMuscla for the treatment of non-dystrophic myotonia, round 2 survey

About you

* 2. What is your name?
Please note that this information will be used for internal tracking purposes only. Your individual responses will remain anonymous.

* 3. Because health resource use (HRU) differs by country, we have separated HRU-related questions for experts who work in the UK and those who work in other countries.

Please reconfirm which country you live and work in? Please note that for experts from outside the UK, we cannot anonymize your answers regarding HRU as only one expert per non-UK country is participating.

- ◯ I work in the UK
- ◯ I work outside the UK (Germany, France or Denmark) and agree for my non-anonymized answers to be used

Healthcare resource utilization in the UK

Proportion of patients requiring resource use per year

In Round 1, we aimed to identify what proportion of patients with NDM make use of healthcare resources, and investigate whether there is a difference between patients who receive best supportive care (BSC) compared with patients who are on treatment with NaMuscla® (mexiletine) in the UK. For the purpose of this survey, BSC is defined as any supportive care that symptomatic adult patients with NDM may receive that does not involve symptom-modifying pharmacological treatment. However, BSC includes, for example, the use of supportive medication (e.g. pain killers), mobility aids, physiotherapy or speech therapy.

Table 2 provides the results of the first round.

While a variety in answers were provided, as can be seen by the large ranges, there was consensus that the proportion of patients requiring day case attendances and wheelchair use would be the same for those receiving BSC versus those treated with NaMuscla (mexiletine).

For the resources for which there was no consensus, the results suggest that on average the proportion of patients requiring each HRU decreases with NaMuscla (mexiletine) use. We would like to explore this in the next questions.

Table 2: Difference in the proportion of patients requiring resource use when treated with BSC vs NaMuscla in the UK (n=5)*

Health resource	BSC – Mean % of patients requiring resource (range)	NaMuscla – Mean % of patients requiring resource (range)	Difference (%)	BSC:NaMuscla ratio**
Physiotherapy	39.0 (15–60)	23.0 (5–60)	-16.0	1.7
Occupational therapist	15.0 (5–25)	6.0 (0–15)	-9.0	2.5
Speech therapy	5.0 (0–15)	1.0 (0–5)	-4.0	5.0
Day case attendances*	60.0 (0–100)	60.0 (0–100)	0.0	0
Use of wheelchair	4.2 (0–20)	4.2 (0–20)	0.0	0
Use of walking stick	7.2 (0–30)	2.2 (0–10)	-5.0	3.3
Use of walking frame	0.4 (0–2)	0.0 (0–0)	-0.4	0
Hospital admission for fracture	3.0 (0–10)	1.0 (0–5)	-2.0	3.0
Overall BSC:NaMuscla ratio, including all resources				**1.9**
Overall BSC:NaMuscla ratio excluding day case attendances and wheelchair use (resources for which there was consensus that use would be the same for both groups)				2.6

Key: BSC, best supportive care.

Notes: *The green cells indicate resources for which there was consensus that there is no difference between patients receiving BSC and those treated with NaMuscla. **The ratio is calculated by dividing the mean proportion of patients receiving BSC that require a resource by the mean proportion of patients treated with NaMuscla that require a resource.

4. We suspect that the range of HRU reflects the heterogeneity of the condition. In addition, resource use will always differ to some extent in different institutions. We will therefore not follow up on the means and ranges provided in Table 2. However, if you wish to provide any additional information, please use the text box below:

* 5. It is clear from the responses that, except for day case attendances and wheelchair use, the proportion of patients requiring each HRU **on average** decreases with NaMuscla (mexiletine) use.

Please complete the table below by indicating if you agree or disagree with the following statement: ‘although there is variation in HRU, the proportion of patients who require this resource will **on average** be lower for patients who are treated with NaMuscla (mexiletine) compared with patients receiving BSC **in the UK’**. If you disagree, please explain your answer.

	Agree	Disagree
**Physiotherapy**	○	○
Please explain your answer here		
**Occupational therapist**	○	○
Please explain your answer here		
**Speech therapy**	○	○
Please explain your answer here		
**Use of walking stick**	○	○
Please explain your answer here		
**Use of walking frame**	○	○
Please explain your answer here		
**Hospital admission for fracture**	○	○
Please explain your answer here		

* 6. Please refer to the last column of Table 2. Looking at the overall difference in mean resource use across all resources, the proportion of patients with NDM requiring resource use is **on average** 1.9 times higher for patients who receive BSC, compared with those treated with NaMuscla (mexiletine).

Do you feel that this is a reasonable reflection of the difference in the UK?

- ◯ Yes
- ◯ No

If 'no' please explain your answer:

Additional identified health resources in the secondary- and other care settings

The panellists were asked if any items of resource use in the secondary or other care settings were missing from the tables above. Table 3 presents the additional resources that were identified by the panel.

Table 3: Additional identified resources in the secondary- and other care settings

Identified resource (number of respondents who identified resource)
Secondary care setting<br>NDM family care advisor (n=1)<br><br>Other care setting<br>Third-sector financial advice (Muscular Dystrophy Advocacy Officer) (n=1)

7. We do not require any further information on this, but if you wish to add any additional information that you feel will add value, please do so in the box below:

Frequency of health resource use per year

In Round 1 of the Delphi panel, we explored the frequency of healthcare visits for those patients that make use of a resource. We also investigated if there are any differences between patients who received BSC versus those who are treated with NaMuscla (mexiletine) in the UK. Table 4 provides the results of the first round.

No consensus was achieved for this question. However, the results suggest that on average the frequency of healthcare visits by patients who make use of a particular resource decreases with NaMuscla (mexiletine) use. We would like to explore this in the next questions.

Table 4: Difference in the mean number of visits per year for adult patients with NDM, comparing those who receive BSC vs those treated with NaMuscla (mexiletine) in the UK

Health resource	BSC – Mean number of annual visits (range)	NaMuscla – Mean number of annual visits (range)	Difference	BSC : NaMuscla ratio *
Physiotherapy	5.0 (2–9)	4.0 (0–9)	-1.0	1.3
Occupational therapist	2.2 (1–5)	1.2 (0–4)	-1.0	1.8
Speech therapy	1.2 (0–5)	0.4 (0–2)	-0.8	3.0
Day case attendances	1.0 (0–2)	0.8 (0–2)	-0.2	1.3
Overall BSC:NaMuscla ratio				**1.8**

Key: BSC, best supportive care.
Note: * The ratio is calculated by dividing the mean number of annual visits of patients receiving BSC that require a resource by the mean number of annual visits of patients treated with NaMuscla that require a resource.

8. If you have any additional information you would like to share on the mean numbers and ranges provided in Table 4, please use the text box below:

* 9. Please complete the table below by indicating if you agree or disagree with the following statement: ‘although there is variation in the number of healthcare visits among patients with NDM, the number of visits of patients who require this resource will **on average** be lower for patients who are treated with NaMuscla (mexiletine) compared with patients receiving BSC **in the UK’**

	Agree	Disagree
**Physiotherapy**	◯	◯
Please explain your answer here		
**Occupational therapist**	◯	◯
Please explain your answer here		
**Speech therapy**	◯	◯
Please explain your answer here		
**Day case attendances**	◯	◯
Please explain your answer here		

* 10. Please refer to the last column of Table 4. Looking at the difference in mean number of healthcare visits for all resources, the number of visits of patients with NDM requiring resource use is **on average** 1.8 times higher for patients who receive BSC, compared with those treated with NaMuscla (mexiletine).

Do you feel this is a reasonable reflection of the difference **in the UK**?

◯ Yes

◯ No

If 'no' please explain your answer:

Mental health support

In Round 1 of the Delphi panel, we considered the proportion of adult patients with NDM who would require (any form of) mental health support. Table 5 shows the results.

While a range of proportions were given, there was consensus among all UK experts who completed the Delphi panel that the proportion of patients requiring mental health support is lower for patients who are treated with NaMuscla (mexiletine) compared with those receiving BSC.

Table 5: Comparison of the proportion of patients treated with BSC vs NaMuscla (mexiletine) who require mental health support in the UK

BSC – Mean proportion of patients (range)	NaMuscla (mexiletine) – Mean proportion of patients (range)	Mean difference	BSC:NaMuscla ratio*
38 (20–60)	14 (5–20)	-24	**2.7**

Key: BSC, best supportive care
Note: * The ratio is calculated by dividing the mean proportion of patients receiving BSC that require mental health support by the mean proportion of patients treated with NaMuscla that require mental health support.

11. If you have any additional information you would like to share on the mean numbers and ranges provided in Table 5, please use the text box below:

* 12. Please refer to the last column of Table 5. Looking at the proportion of patients with NDM who require mental health support, the need for mental health support is **on average** 2.7 times higher for patients who receive BSC, compared with those treated with NaMuscla (mexiletine).

Do you feel this is a reasonable reflection of the difference **in the UK**?

- Yes
- No

If 'no' please explain your answer:

The panel was also asked what mental health resources patients with NDM may use. Table 6 gives an overview of the identified resources.

Table 6: Identified mental health resources in the UK (number of respondents who identified this resource)

- Psychiatric referral (not further specified) (n=1)
- Counselling (not further specified) (n=1)
- Neuropsychologist (n=1)
- General practitioner (n=1)
- UK NHS 'Healthy Minds' (n=1)
- UK NHS adult Improving Access to Psychological Therapies (IAPT) service (n=1)

As the results of the Delphi panel suggest that mental health support is required by a proportion of patients with NDM, we would like to explore in more detail what proportion of patients makes use of each individual mental health resource and how often. Please note that charities are currently not included as there are no direct cost to the NHS associated with these resources.

Proportion of patients requiring mental health resource use

* 13. From your experience, of the patients who receive mental health support, please provide an estimation of **the percentage** of adult patients with NDM, who receive **BSC**, that would use each type of resource related to mental health support in the table below. Please ensure that the values are between 0-100%. [All rows require an answer]

Neuropsychologist	
General practitioner (mental health support related visits)	
Psychiatrist	
Psychologist	

* 14. From your experience, of the patients who receive mental health support, please provide an estimation of **the percentage** of adult patients with NDM, who are treated with **NaMuscla (mexiletine)**, that would use each type of resource in the table below. Please ensure that the values are between 0-100%. [All rows require an answer]

Neuropsychologist	
General practitioner (mental health support related visits)	
Psychiatrist	
Psychologist	

15. If there are any resources related to mental health support missing from the table above, please list them here and indicate what **percentage** of patients treated with NaMuscla (mexiletine) and BSC would use them:

Frequency of mental health resource use

* 16. Of the adult patients with NDM who receive **BSC** who make use of a resource for mental health, please provide an estimation of **how often** that patient would use the resource per year (in numbers). [All rows require an answer, only numbers allowed].

Neuropsychologist

General practitioner (mental health support related visits)

Psychiatrist

Psychologist

* 17. Of the adult patients with NDM who treated with **NaMuscla (mexiletine)** who make use of a resource for mental health, please provide an estimation of **how often** that patient would use the resource per year (in numbers). [All rows require an answer, only numbers allowed]

Neuropsychologist

General practitioner (mental health support related visits)

Psychiatrist

Psychologist

18. If there are any resources related to mental health support missing from the table above, please list them here and indicate **how often** patients treated with NaMuscla (mexiletine) and BSC would use these per year:

Healthcare resource utilization in France, Germany and Denmark

Proportion of patients requiring resource use per year

In Round 1 of the Delphi panel, we explored the proportion of patients who utilize different health resources and investigated if there are any differences between patients who receive BSC versus those who receive NaMuscla (mexiletine). The results are shown in Table 7.

Table 7: Difference in the proportion of patients requiring resource use when treated with BSC vs NaMuscla in France, Germany and Denmark.

Health resource	France (%)			Germany (%)			Denmark (%)		
	BSC	Nam	Difference	BSC	Nam	Difference	BSC	Nam	Difference
Physiotherapy	40	10	-30	100	100	0	25	25	0
Occupational therapist	20	10	-10	20	20	0	15	15	0
Speech therapy	30	5	-25	20	20	0	5	0	-5
Day case attendances	100	50	-50	100	100	0	5	0	-5
Use of wheelchair	10	0	-10	1	1	0	0	5	+5
Use of walking stick	20	10	-10	10	10	0	10	0	-10
Use of walking frame	10	5	-5	1	1	0	0	0	0
Hospital admission for fracture	5	2	-3	1	1	0	0	0	0

Key: BSC, best supportive care; Nam, NaMuscla (mexiletine).

19. We do not require any further information on this, but if you wish to add any additional information that you feel will add value, please do so in the box below:

Additional identified health resources in the secondary- and other care settings

The panellists were asked if any items of resource use in the secondary or other care settings were missing from the tables above. One additional resource was reported: ‘social care’.

20. We do not require any further information on this, but if you wish to add any additional information, please do so in the box below:

Frequency of health resource use per year

In Round 1 of the Delphi panel, we also explored the frequency of patients who utilize different health resources and investigate if there are any differences between patients who received BSC versus those who receive NaMuscla (mexiletine). Table 8 shows the results.

Table 8: Difference in frequency of resource use per year for adult patients with NDM, comparing those who are treated with BSC vs NaMuscla (mexiletine) in France, Germany, and Denmark

Health resource	France			Germany			Denmark		
	BSC	Nam	Difference	BSC	Nam	Difference	BSC	Nam	Difference
Physiotherapy	30	5	-25	24	24	0	40	40	0
Occupational therapist	10	2	-8	12	12	0	6	6	0
Speech therapy	15	2	-13	12	12	0	6	0	-6
Day case attendances	5	1	-4	4	4	0	6	0	-6

Note: Whenever a 0 is presented, this reflects that the expert reported that they expect 0 HCP visits for that resource.
Key: BSC, best supportive care; Nam, NaMuscla (mexiletine).

21. We do not require any further information on this, but if you wish to add any additional information about the table above that you feel will add value, please do so in the box below:

Mental health support

In Round 1 of the Delphi panel, we considered the proportion of adult patients with NDM who would require (any form of) mental health support. The results are presented in Table 9.

Table 9: Comparison of the proportion of patients treated with BSC vs NaMuscla (mexiletine) who require mental health support in France, Germany and Denmark

Proportion of patients requiring mental health support	France	Germany	Denmark
Patients treated with **BSC**	20	0	5
Patients treated with **NaMuscla (mexiletine)**	5	0	5
Difference	-15	0	0

22. We do not require any further information on this, but if you wish to add any additional information about the table above that you feel will add value, please do so in the box below:

Duration of healthcare visits

In Round 1 of the Delphi panel, you were asked to provide an estimate of how long each of the below visits will take (in minutes). Results are shown in Table 10.

Table 10: Average duration of health care visits

Health resource	France	Germany	Denmark
Physiotherapy	20 minutes	30 minutes	30–60 minutes
Occupational therapist	45 minutes	30 minutes	1–3 hours
Speech therapy	30 minutes	30 minutes	30 minutes
Day case attendances	30 minutes	30 minutes	1–3 hours

23. We do not require any further information on this, but if you wish to add any additional information that you feel will add value, please do so in the box below:

Delphi panel to support the submission of NaMuscla for the treatment of non-dystrophic myotonia, round 2 survey

Treatment with NaMuscla (mexiletine)

Average amount of capsules taken

In Round 1 of the Delphi panel, we asked the panellists how many capsules an adult patient with NDM who receives NaMuscla (mexiletine) will take on average per day in the long-term. Figure 1 presents the results.

There was consensus that on average, in the long-term, two capsules of NaMuscla would be taken per day by adult patients with NDM. We would like to explore if this is the same for adults aged >65 years old.

Figure 1: Mexiletine treatment, average number of capsules taken per day

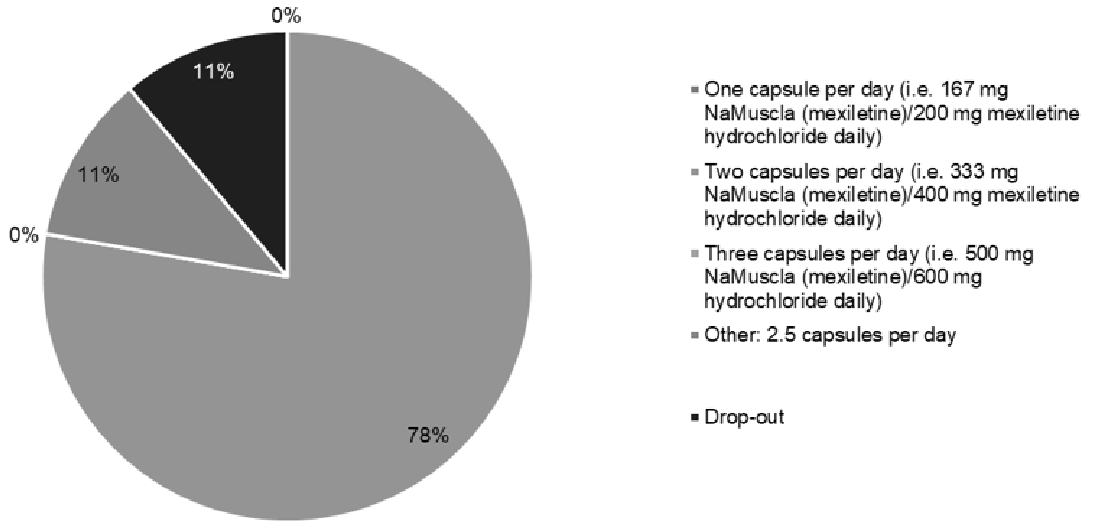

* 24. Would patients aged >65 years old be treated the same, with an average daily dose of two capsules NaMuscla (mexiletine) per day?

- ◯ Yes
- ◯ No, the average daily dose would be **higher** than two capsules per day
- ◯ No, the average daily dose would be **lower** than two capsules per day

If 'no', please explain what average daily dose you would expect patients aged >65 years old to take:

Eligibility criteria for treatment

In Round 1 of the Delphi panel, we asked when the panel would consider adult patients with NDM to be eligible for NaMuscla (mexiletine) treatment. Results are show in Figure 2.

There was consensus among the panellists that they will consider adult patients with NDM to be eligible for NaMuscla (mexiletine) treatment if they have:

- Genetically confirmed NDM, (n=8, 89%)
- Symptoms severe enough to impact their daily lives (n= 7, 78%)
- A normal cardiac exam as performed by a cardiologist, including electrocardiogram (EKG) and cardiac ultrasound (n= 7, 78%).

No consensus was reached for the other criteria.

Figure 2: Criteria to select patients for mexiletine treatment

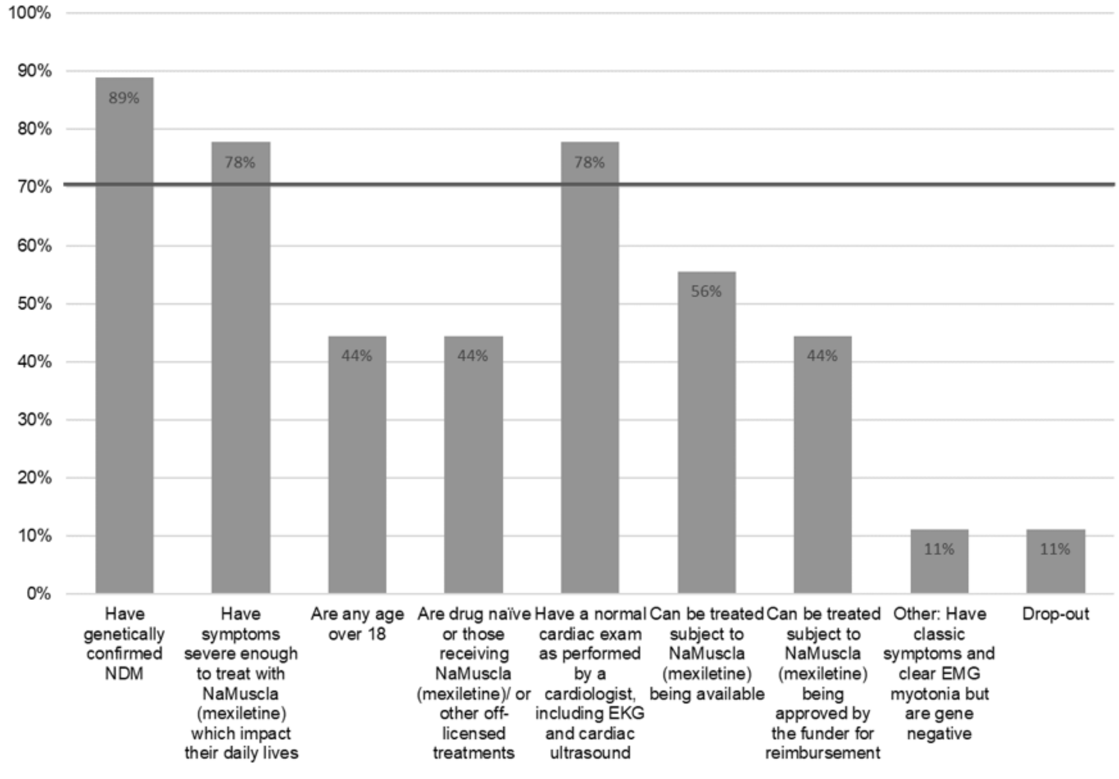

Key: EKG, electrocardiogram; EMG, electromyography; NDM, non-dystrophic myotonia
Note: The red line indicates the 70% consensus threshold.

25. We do not require any further information on this, but if you wish to add any additional information, please do so in the text box below:

Delphi panel to support the submission of NaMuscla for the treatment of non-dystrophic myotonia, round 2 survey

Instruments to measure quality of life and disease severity

In Round 1 of the Delphi panel, we presented the absolute change in median stiffness visual analogue (VAS) score and INQoL score from the MYOMEX trial, which compared patients who receive BSC with patients who are treated with NaMuscla (mexiletine). We asked the panellists if the changes from baseline are a clinically important difference. Results are shown in Figure 3.

There was a consensus that both the change in median stiffness VAS score and median INQoL score are a clinically important difference.

Figure 3: Clinically important difference in the absolute change in median stiffness VAS score (left) and INQoL score (right)

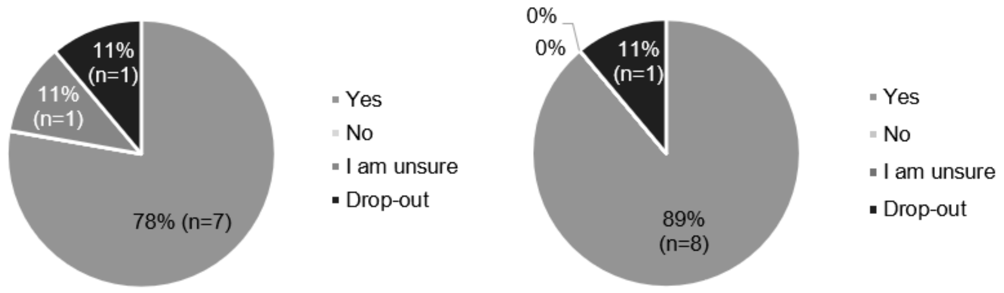

Key: INQoL, Individualised Neuromuscular Quality of Life Questionnaire; VAS, visual analogue scale

26. As there was a consensus, we do not require any further information on this. We would like to thank everyone who provided additional comments on this question. If you wish to add any additional information, please use the box below:

Matching exercise

Matching domains of INQoL to domains of the EQ-5D

The National Institute for Health and Care Excellence (NICE) requires manufacturers to express the health effects of their products in quality-adjusted life years, which considers both quality of life (QoL) as well as length of life. NICE prefers using the EQ-5D as a measure for quality of life. While EQ-5D is a generic instrument and therefore less specific than disease-specific instruments such as INQoL, a key benefit is that it allows NICE to compare health effects across different diseases.

In the MYOMEX trial, QoL data was captured using the INQoL and VAS in patients with NDM. To meet NICE's requirement and support the economic model of NaMuscla, we aimed to explore what domains of INQoL match to domains of EQ-5D in Round 1 of the Delphi panel.

We have received feedback from most panellists that this was a challenging exercise. This was mainly because the two questionnaires are very different, but also because signs and symptoms of the condition may impact different patients in different ways, and domains such as 'mobility' are influenced by multiple underlying factors.

While we understand the difficulties of this question and its limitations, we want to explore the current findings in more detail.

Figure 4 shows the results of Round 1.

There was consensus that:

- **'Usual activities' from EQ-5D maps best to 'the things you do – leisure and work activities' from INQoL**
- **'Pain/discomfort' from EQ-5D maps best to 'your pain' from INQoL.**
- **'Anxiety/depression' from EQ-5D maps best to 'how you feel/emotions' from INQoL**

No consensus was reached for the 'mobility' and 'self-care' domains. We would like to follow-up this in the next questions.

Figure 4: Mapping domains of INQoL to EQ-5D

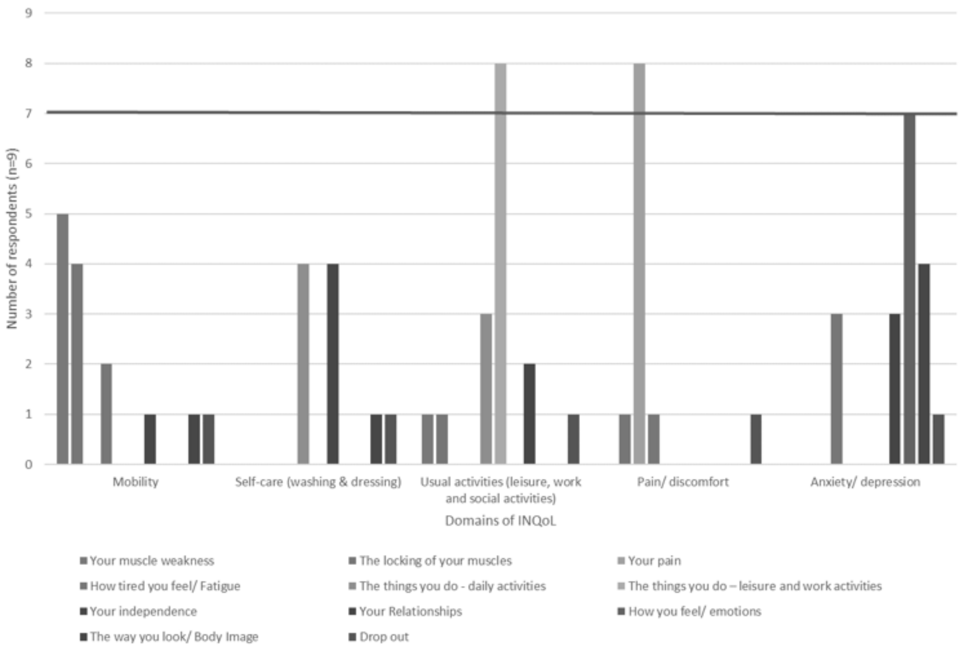

Key: INQoL, Individualised Neuromuscular Quality of Life Questionnaire
Note: the red line indicates the 70% consensus threshold.

When answering the below questions, please refer to the INQoL and EQ-5D that were sent in the pre-read materials.

* 27. The domains 'your muscle weakness' and 'the locking of your muscles' of INQoL were most frequently mapped to the 'mobility' domain of EQ-5D. The questions associated with these domains of INQoL are listed below. Please tick the box with the question of INQoL that best maps to the 'mobility' domain of EQ-5D:

- ◯ Do you have any muscle weakness due to your muscle condition? (yes/no)
- ◯ How much muscle weakness would you say you have in the muscles affected by your condition? (rating: very little–an extreme amount)
- ◯ Does your muscle weakness cause difficulties in your life at the moment? (rating: none at all–an extreme amount)
- ◯ How important to you are any difficulties caused by your muscle weakness? (rating: not at all important–extremely important)
- ◯ Do you have any 'locking' (seizing up) of your muscles as a result of your muscle condition? (yes/no)
- ◯ How much muscle 'locking' would you say you have at the moment? (rating: very little–an extreme amount)
- ◯ Does the 'locking' of your muscles cause difficulties in your life at the moment? (rating: none at all–an extreme amount)
- ◯ How important to you are any difficulties caused by the 'locking' of your muscles? (rating: not at all important–extremely important)

* 28. The domains ‘the things you do – daily activities’ and ‘your independence’ of INQoL were most frequently mapped to the ‘self-care (washing and dressing)’ domain of EQ-5D. The questions associated with these domains of INQoL are listed below. Please tick the box with the question of INQoL that best maps to the ‘self-care (washing and dressing)’ domain of EQ-5D:

- ◯ At the moment, does your muscle condition affect your ability to do the following activities: daily activities (for example, washing, dressing and housework)? (rating: not at all–extremely)
- ◯ In the face of my condition, my ability to do all the things I want to do is? (rating: exactly as I would like it to be–the worst it could possibly be)
- ◯ How important to you is the effect of your muscle condition on your ability to do all the things you want to do? (rating: not at all important–extremely important)
- ◯ At the moment, how much help do you need from other people in carrying out your activities (for example, daily activities and going out)?
- ◯ In the face of my condition, my level of independence is? (rating: exactly as I would like it to be–the worst it could possibly be)
- ◯ How important to you is the effect of your muscle condition on your level of independence? (rating: not at all important–extremely important)

Matching exercise: matching items of INQoL to domains of the EQ-5D

In Round 1 of the Delphi Panel, we set out to map items of INQoL to domains of EQ-5D. Only Half of the panellists answered this question and therefore no consensus could be reached, the results are shown in Table 11.

29. Given the complexity of this exercise, we believe that a Delphi panel is not the best method to explore this in more detail. We will therefore not follow-up on this question. However, if you wish to provide any additional information that you feel would add value, please use the text box below:

Table 11: Matching items of INQoL to domains of EQ-5D (n=4)

Domain of EQ-5D	Matching item(s) of INQoL
Mobility	1A (n=1), 1B (n=1), 2A (n=1), 4A (n=1)
Self-care (washing and dressing)	5AI (n=2), 6A (n=2)
Usual activities (leisure, work and social activities)	1B (n=1), 5AI (n=1), 5AII (n=4), 5AIII (n=1), 5BI (n=1), 5BII (n=1)
Pain and discomfort	2AI (n=1), 3A (n=3), 3B (n=1)
Anxiety and depression	4A (n=1), 7AI (n=1), 7AII (n=1), 7AIII (n=1), 7AIV (n=2), 8AI (n=2), 8AII (n=2), 8AIII (n=1), 8AIV (n=1), 8BI (n=2), 9BI (n=1), 9BII (n=1)
**Key:** INQoL, Individualised Neuromuscular Quality of Life Questionnaire<br>**Note**: whenever an expert did not specify what sub-element of an item maps best to EQ-5D, it was assumed that they felt all sub-elements are applicable. For example, the answer ‘8A’ would be counted as 8AI-8AIV.	

Ranking domains of INQoL based on their impact on patient QoL

Panellists were asked to rank domains of INQoL based on their impact on patient QoL (ranging from 1 – high impact, to 10 – low impact). The results are shown in Table 12 and Figure 5.

The responses to this question varied widely. However, what could be observed is that in general:

- ‘the locking of your muscles’, ‘your muscle weakness’, ‘your pain’, and ‘how you feel/emotions’ were most frequently considered impactful (modes of 1, 2, 2, and 3, respectively).
- ‘the way you look/body image’ and ‘your relationships’ were most frequently considered less impactful (modes of 10 and 8, respectively).

30. We suspect that the variety in answers reflects the heterogeneity of the condition as well as individual perceptions of QoL. We will therefore use the information as it is and have decided not follow-up on this further. However, if you wish to provide any further information, please use the text box below:

Table 12: Ranking of domains of INQoL that impact QoL the most

Domain of INQoL	Mode	Median	Min	Max
Your muscle weakness	2	5	1	10
The locking of your muscles	1	1.5	1	8
Your pain	3	3	1	6
How tired you feel/fatigue	4	4.5	1	10
The things you do – daily activities	6	5.5	3	9
The things you do – leisure and work activities	7	6.5	2	10
Your independence	5	5	4	7
Your relationships	8	8	3	9
How you feel/emotions	2	3.5	1	9
The way you look/body image	10	9.5	8	10

Key: INQoL, Individualised Neuromuscular Quality of Life Questionnaire; QoL, quality of life

Figure 5: Ranking domains of INQoL based on their impact on patient QoL

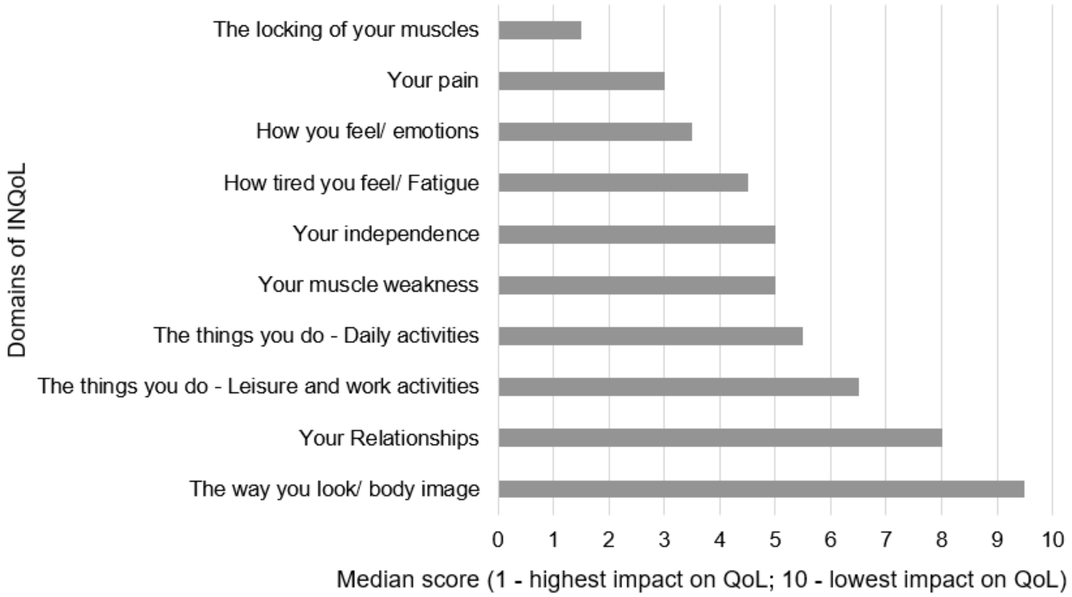

Key: INQoL, Individualised Neuromuscular Quality of Life Questionnaire; QoL, quality of life

Natural history

Proportion of patients experiencing a change in their QoL

In Round 1, we compared what proportion of patients with NDM will experience an increase, decrease or no change at all in their QoL over their lifetime when treated with BSC or NaMuscla (mexiletine). The results are shown in Table 13 and Figure 6.

While a range of answers were provided, the results suggest that on average more patients who are treated with NaMuscla (mexiletine) are expected to experience a QoL increase, compared with patients who only receive BSC. This subsequently results in a lower proportion of patients who are treated with NaMuscla (mexiletine) who experience a decrease or no change in their QoL compared with those who only receive BSC. We would like to explore this in more detail.

Table 13: Difference in proportion of adult patients with NDM with BSC vs NaMuscla (mexiletine) who will experience an overall increase, decrease or no change in the disease-related QoL over their lifetime

Estimates	BSC – Mean (range)	NaMuscla – Mean (range)	Difference (%)	BSC:NaMuscla ratio*
Increase	28 (0–100)	59 (0–100)	+31	0.5
Decrease	36 (0–90)	17 (0–70)	-19	2.1
No change	36 (0–90)	24 (0–50)	-12	1.5

Key: BSC, best supportive care

Note: *The ratio is calculated by dividing the mean proportion of patients receiving BSC by the mean proportion of patients treated with NaMuscla.

Figure 6: Difference in proportion of adult patients with NDM who will experience an overall increase, decrease or no change in the disease-related QoL over their lifetime comparing patients treated with BSC with patients treated with NaMuscla (mexiletine)

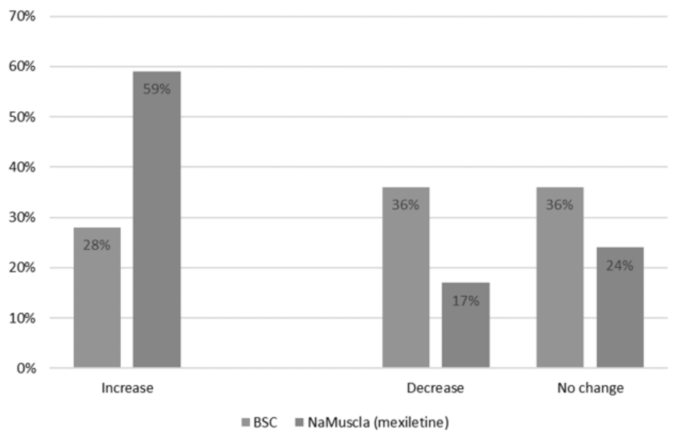

Key BSC, best supportive care; NDM, non-dystrophic myotonia; QoL, quality of life

* 31. Please indicate if you agree or disagree with the following statement: '**on average** the proportion of patients who experience a QoL increase over their lifetime will be higher for those who are treated with NaMuscla (mexiletine) compared with patients receiving BSC'

- Agree
- Disagree

If your disagree, please explain your answer here:

Annual rate at which QoL decreases

In a follow-up question, we explored the annual rate at which QoL decreases for some patients and whether this is different for patients who receive BSC compared with those receiving NaMuscla (mexiletine) (Figure 7).

Figure 7: Difference in the annual rate at which disease-related QoL decreases over time for patients who receive BSC compared with patients receiving NaMuscla (mexiletine) (n=9)

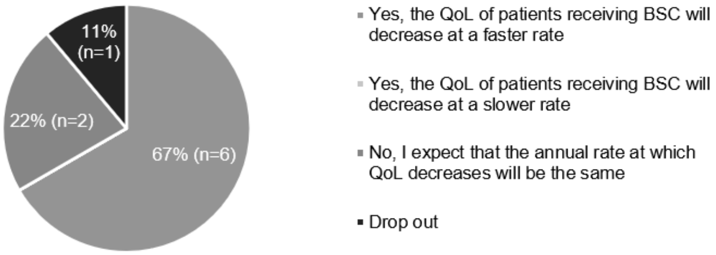

Key: BSC, best supportive care; QoL, quality of life

No consensus was reached for this question in the first round.

Six experts noted that, for those patients who experience a QoL decrease over their lifetime, the QoL of patients who only receive BSC will decrease at a faster rate, compared with patients who are treated with NaMuscla (mexiletine). The following explanations were given.

- ‘QoL decline in some mexiletine non-responders patients may be lower considering a pharmacological treatment is administered as compared to no drug.’
- ‘Limited side effects, benefit to symptoms if taking treatment’
- ‘This is an impression but is partly based on more patients having to give up activities or work if only receiving BSC.’
- ‘Patient who use Mexiletine generally decline much more slowly than NDM patients who receive only physio and occupational therapy’

Two experts indicated that QoL will decrease at the same rate in patients who receive BSC compared with those receiving NaMuscla (mexiletine). One explanation was given:

- ‘Stone et al may have recorded a self-reported increase in symptoms over time but that does not mean that QoL will track the change in symptom severity. Indeed, evidence in other fields may suggest that QoL stabilizes over time especially in response to slow changes in symptoms. I would want to critically review the evidence that you have for decline in QoL over time. I don't think we have any evidence on any rate of any decline that would allow anybody to accurately answer this question.’

We would like to follow-up on this, in the current Delphi round.

* 32. Considering the above explanations, please indicate if, for those patients who experience a decline in their QoL, you expect the **annual rate** at which disease-related QoL decreases over time will be different for patients who receive BSC compared with patients treated with NaMuscla (mexiletine)?

- ◯ No, I expect that the annual rate at which QoL decreases will be the same
- ◯ Yes, I expect that the QoL of patients receiving BSC will decrease at a faster rate annually compared with patients treated with NaMuscla (mexiletine)
- ◯ Yes, I expect that the QoL of patients receiving BSC will decrease at a slower rate annually compared with patients treated with NaMuscla (mexiletine)

* 33. The Evidence Review Group (ERG) at NICE, and the company consider scenarios of different annual rates at which QoL decreases over time.

Please consider the options below and tick the box which you feel is most appropriate. “I expect the annual rate at which QoL decreases over time for patients receiving BSC compared with patients treated with NaMuscla (mexiletine) to be”:

- ◯ 0% (i.e. there is no difference in the annual rate at which QoL declines for patients receiving BSC compared with patients treated with NaMuscla)
- ◯ 5%
- ◯ 10%
- ◯ 15%
- ◯ 20%
- ◯ 25% (i.e. the annual rate at which QoL decreases of patients receiving BSC is 25% faster compared with patients treated with NaMuscla)
- ◯ Other, please specify:

Caregiver quality of life

In Round 1, panellists were asked about the impact on caregiver QoL and to compare whether there are any differences between caregivers of patients who receive BSC only, compared with those who are treated with NaMuscla (mexiletine). The results are shown in Figure 8 and Figure 9. In addition, panellists provided explanations for their answers, as shown in Table 14.

No consensus was reached for this question. However, overall, a shift could be observed: more panellists found that caregivers of patients treated with NaMuscla (mexiletine) are 'not impacted' on aspects of QoL, compared with caregivers of patients receiving BSC only. In addition, none of the experts felt that aspects of the caregivers QoL are 'significantly' impacted when a patient is treated with NaMuscla (mexiletine).

Figure 8: Impact on caregiver QoL – BSC

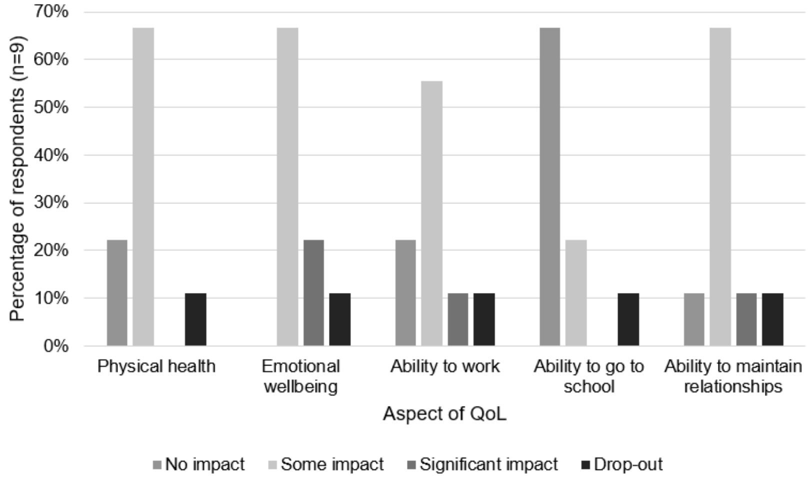

Key: BSC, best supportive care; QoL, quality of life

Figure 9: Impact on caregiver QoL – NaMuscla (mexiletine)

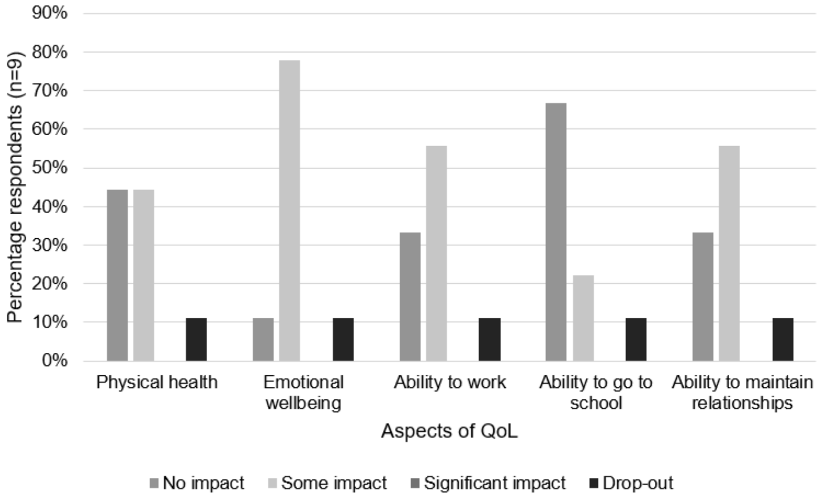

Key: QoL, quality of life

Table 14: Explanations given by panellists for why caregivers might experience 'some' or 'significant' impact on aspects of their QoL

Impact on caregivers who care for patients who receive BSC
'Social consequences related to overt stiffness, reduced mobility.'
'Can be difficult to maintain relationship with person in pain and needing support, may need help with ADLs.'
'It will depend on degree and amount of support required. Most NDM patients are not severely disabled but a small minority are. There is a significant impact on shared activities but most NDM patients will be able to be left to care for themselves for most of the time. Physical dependence is likely to be less than psychological dependence on others.'
'Some impact is only seen in caregivers to patients with very severe disabling myotonia. In these cases, caregivers may cut down on work hours and may be psychologically affected.'
'Burden of care is dependent on how self-caring and independent the patient is and how well they cope.'
'The physical health, emotional well-being and ability to maintain relations of a caregiver will all be impacted due to the additional amount of time that they have to give over to the person that they're caring for. Chores are not necessarily split evenly, and the carer feels guilty for being unable to maintain relationships outside with other people.'
**Impact on caregivers who care for patients who are treated with NaMuscla (mexiletine)**
'Likely to be improved but some care and support still needed. Boundaries of question too broad to reflect effect.'
'Same response as above, although fewer caregivers likely would be involved.'
'Regardless of symptom relief I imagine that there will be some residual symptoms but also that even if symptom free there may be a burden being in a relationship with anyone who has a long term condition on long term treatment. I expect this would be more psychological and emotional.'

34. We do not require any further information on this. However, if you wish to provide any additional information, please use the text box below:

Delphi panel to support the submission of NaMuscla for the treatment of non-dystrophic myotonia, round 2 survey

End

This is the end of the survey, thank you for participating.

If you have any questions or comments, please do not hesitate to contact Marieke Schurer (Senior Insight Analyst, BresMed) via: mschurer@bresmed.com. Alternatively, please write your comments or questions in the text box below.

35. If you have any comments on the survey, please write them here:

Delphi panel to support the submission of NaMuscla for the treatment of non-dystrophic myotonia, round 2 survey

End of survey

Thank you for your response.

Please contact Marieke Schurer (mschurer@bresmed.com) to discuss any concerns you may have about this study or our adverse event reporting obligations before continuing the survey.

Delphi panel to support the submission of NaMuscla for the treatment of non-dystrophic myotonia, round 2 survey

End of survey
