# Supplementary material for: Improving the understanding of how patients with non-dystrophic myotonia are selected for myotonia treatment with mexiletine (NaMuscla): outcomes of treatment impact using a European Delphi panel
Source: BMC Neurol. 2021 Dec 1;21:467. doi: 10.1186/s12883-021-02491-3 (PMC8633892; doi:10.1186/s12883-021-02491-3)
Supplement: Supplementary file 3 — Additional file 3. [file 12883_2021_2491_MOESM3_ESM.pdf]

Title: Improving the understanding of how patients with non-dystrophic myotonia are selected for myotonia treatment with mexiletine (NaMuscla): outcomes of treatment impact using a European Delphi panel

Authors: Ann-Marie Chapman[1], Marieke Schurer[2*], Laure Weijers[2], Amer Omar[3], Hiba Lee[4], Alla Zozulya-Weidenfeller[3], Crispin Ellis[5], Shaneil Sonecha[5], Christiane Schneider-Gold[6]

***Correspondence:** mschurer@bresmed.com

[1] BresMed Health Solutions Ltd, Sheffield, UK

[2] BresMed Netherlands BV, Utrecht, The Netherlands

[3] Lupin Atlantis Holdings SA, Zug Switzerland

[4] AGH Partners Ltd, London, UK

[5] Lupin Healthcare Ltd, Slough, UK

[6] St. Josef-Hospital, Bochum, Germany

Selection criteria Delphi panellists

Selection criteria for the UK experts

- The participant must work in one of the 18 neuroscience specialist centres as determined by NHS England, or work in one of the main centres in Scotland or Wales.
- The centre should be known to treat at least five NDM patients (quantifiable expertise).
- The participant must be known as a neurology expert in the treatment of NDM patients (qualitative expertise), who Lupin believes would have the knowledge and capability to answer the questions posed in the Delphi survey.
- The participant must be previously responsive to a Lupin invitation, to avoid irritation to any potential expert who would be highly unlikely to engage.

Selection criteria for experts from outside the UK

Experts from outside the UK must:

- Be a key opinion leader in the field:
  - Internationally recognized by peers as expert in the fields of Myotonia
  - Members of neurology societies and neuromuscular research societies
- Have proven international expertise in the field of NDM, as demonstrated through:
  - Experience in clinical research as well as management of patients with NDM,
  - Publications, and
  - Experience as an advocate and speaker on relevant conferences.
